# The NF-YC–RGL2 module integrates GA and ABA signalling to regulate seed germination in *Arabidopsis*

**DOI:** 10.1038/ncomms12768

**Published:** 2016-09-14

**Authors:** Xu Liu, Pengwei Hu, Mingkun Huang, Yang Tang, Yuge Li, Ling Li, Xingliang Hou

**Affiliations:** 1 Key Laboratory of South China Agricultural Plant Molecular Analysis and Genetic Improvement, South China Botanical Garden, Chinese Academy of Sciences, Guangzhou 510650, China; 2 Guangdong Provincial Key Lab of Biotechnology for Plant Development, College of Life Sciences, South China Normal University, Guangzhou 510631, China; 3 University of the Chinese Academy of Sciences, Beijing 100049, China

## Abstract

The antagonistic crosstalk between gibberellic acid (GA) and abscisic acid (ABA) plays a pivotal role in the modulation of seed germination. However, the molecular mechanism of such phytohormone interaction remains largely elusive. Here we show that three *Arabidopsis* NUCLEAR FACTOR-Y C (NF-YC) homologues NF-YC3, NF-YC4 and NF-YC9 redundantly modulate GA- and ABA-mediated seed germination. These NF-YCs interact with the DELLA protein RGL2, a key repressor of GA signalling. The NF-YC–RGL2 module targets *ABI5*, a gene encoding a core component of ABA signalling, via specific CCAAT elements and collectively regulates a set of GA- and ABA-responsive genes, thus controlling germination. These results suggest that the NF-YC–RGL2–ABI5 module integrates GA and ABA signalling pathways during seed germination.

## Abstract (web summary)

Crosstalk between gibberellic acid (GA) and abscisic acid (ABA) regulates seed germination. Here the authors show that NF-YC transcription factors can interact with the RGL2 DELLA protein to regulate expression of *ABI5* and therefore modulate ABA- and GA-responsive gene expression.

## Graphical abstract

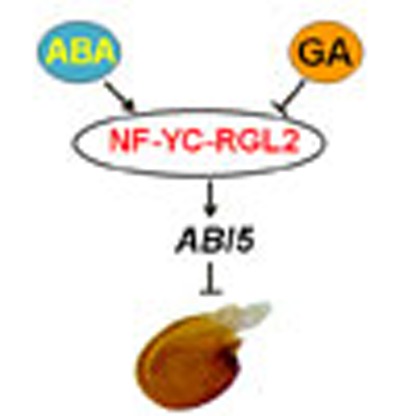

Seed germination is an essential developmental process in the life cycle of higher plants. Plants start with the release of seeds dormancy and launch of germination when the circumstance is favourable for growth, and subsequently, the seedling is established and developing, which serves as the basis for species propagation and agricultural production^1^,^2^. Germination includes a series of sophisticated biochemical reactions tightly regulated by environmental and intrinsic cues such as light irradiation, temperature, water uptake and change of endogenous phytohormone levels, which compose the necessary events to trigger specific signalling for the transition from embryonic to vegetative development^1^,^3^.

Germination process is principally controlled by the phytohormone balance of gibberellic acid (GA) and abscisic acid (ABA), which have antagonistic effects on this vital developmental phase^4^,^5^. GA is one of most important phytohormones that coordinates with a cascade of molecular signalling regulation to promote seed germination^6^. The essential role of GA on germination initiation is best illustrated by previous reports that GA-deficient mutant *ga1* fails to germinate without exogenous GA^7^,^8^. Conversely, ABA counteracts the effect of GA during seed germination by inhibiting water uptake and endosperm rupture rather than testa rupture^9^,^10^. Identification of the ABA-related mutants in *Arabidopsis* has also provided important evidences to reveal the effect of ABA on GA-mediated seed germination. For example, the ABA synthesis-defective mutants *aba1* and *aba2* are able to rescue the non-germinating phenotype of *ga1* (refs 11, 12, 13), supporting the antagonistic roles of ABA and GA during seed germination process.

DELLA proteins serve as the key repressors in GA signalling pathway to modulate plant growth and development. In *Arabidopsis*, five DELLA family members, GA-INSENSITIVE (GAI), REPRESSOR OF *ga1-3* (RGA), RGA-LIKE 1 (RGL1), RGL2 and RGL3, share the conserved DELLA motif and display redundant and distinct roles under the control of GA receptor-mediated degradation^14^,^15^,^16^,^17^,^18^. Among them, RGL2 has been considered as the major negative regulator in the light-dependent seed germination since loss of function of *RGL2* is sufficient to suppress the non-germinating phenotype of the *ga1* mutant^16^,^19^,^20^. In addition, several studies revealed that a bZIP transcriptional factor ABA INSENSITIVE 5 (ABI5), the central ABA signalling component which directly regulates the late embryonic and abundant (LEA) genes including *EM1* and *EM6*, might serve as the final downstream repressor of seed germination in the counterbalance of ABA and GA signals^21^,^22^,^23^. When GA levels are low, the accumulation of RGL2 leads to an increase in endogenous ABA levels by activating the expression of *XERICO* gene that encodes an unknown RING-H2 zinc finger protein involved in ABA synthesis, in turn elevates ABI5 transcription and protein levels, thus inhibiting seed germination^23^,^24^,^25^. Although studies have suggested a significant crosstalk of GA and ABA signalling during seed germination, the detailed mechanism of antagonism between these two phytohormones by which the plants precisely modulate germination remains elusive.

The NUCLEAR FACTORY C proteins (NF-YCs), are structurally characterized by a histone-fold domain (HFD) and closely related to the core histone H2A, functionally act as one subunit of the NF-Y heterotrimer transcriptional factor that specifically recognizes the CCAAT-box in eukaryotes^26^,^27^. In plants, NF-YCs function as important participants in various developmental and stress responses including flowering control^28^,^29^,^30^ and abiotic stress resistance^31^,^32^,^33^,^34^. Recently, studies demonstrated that NF-YCs are also involved in the regulation of phytohormone response^35^,^36^. The diverse roles of NF-YCs, together with those of another two NF-Y subunits NF-YA and NF-YB, imply the widely flexible formation of NF-Y complex that are spatially and temporally regulated by various developmental and growth conditions^27^,^37^.

In this study, we showed that three NF-YC homologues NF-YC3, NF-YC4 and NF-YC9 are engaged redundantly in the suppression of GA-mediated seed germination through directly interacting with the DELLA protein RGL2, a major GA signalling repressor in germination. Further genome-wide transcriptome analysis reveals that the NF-YC–RGL2 module integrates GA and ABA signalling to converge at a set of GA- and ABA-responsive genes. Notably, NF-YCs and RGL2 were showed to directly regulate *ABI5* gene expression regardless of ABA synthesis via recognizing two specific CCAAT elements in the *ABI5* promoter. Consistently, the ABA-mediated germination inhibition is attenuated in the null mutants of *NF-YCs* and *RGL2*, suggesting that NF-YC and RGL2 are required for ABA-mediated seed germination. These findings establish that NF-YCs, together with DELLAs, act as the critical joint modulators to synergistically mediate the antagonism of GA and ABA, providing a new insight into understanding towards phytohormones fine-tuning in seed germination in plants.

## Results

### NF-YC homologues repress GA-mediated seed germination

**Figure 1:**
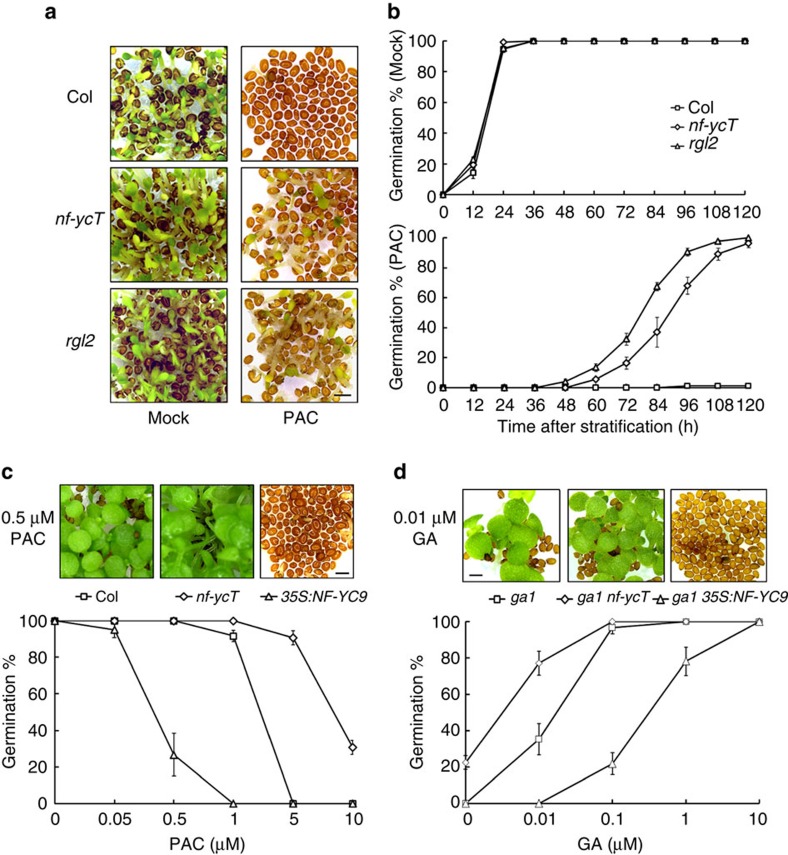
NF-YCs inhibit GA response in seed germination. (**a**) Loss of function of three *NF-YC* homologues or *RGL2* decreases the sensitivity of seeds germination to PAC. Germination phenotypes of *nf-yc3-2 nf-yc4-1 nf-yc9-1* (*nf-ycT*), *rgl2* and the wild-type (Col) seeds were observed at 96 HAS (hour after stratification) on 1/2 MS medium containing 5 μM PAC or mock, respectively. (**b**) Statistic analysis of germination rate in *nf-ycT*, *rgl2* and the wild-type seeds. The seeds were treated as described in **a** and the germination rates were recorded every 12 h until 120 HAS. (**c**) Overexpression of *NF-YC9* seeds increases the sensitivity of seeds germination to PAC. Upper panel shows germination phenotypes of Col, *nf-ycT* and *35S:NF-YC9#1* seeds observed at 120 HAS on 1/2 MS medium containing 0.5 μM PAC. Lower panel shows germination rates of these seeds in response to different concentrations of PAC that were recorded at 120 HAS. (**d**) Loss of function of NF-YCs or overexpression of *NF-YC9* affects the sensitivity of seeds in *ga1* background to GA. Upper panel shows germination phenotypes of *ga1*, *ga1 nf-ycT* and *ga1 35S:NF-YC9* seeds observed at 120 HAS on 1/2 MS medium containing 0.01 μM GA. Lower panel shows germination rate of these seeds in response to different concentrations of GA that were recorded at 120 HAS. All data represent mean±s.d. of at least 100 seeds. Scale bar, 1 mm.

We previously revealed that NF-Y complex regulates flowering time under GA pathway^36^. As GA-mediated molecular response is critical for seed germination, to investigate the role of *Arabidopsis NF-Y* genes in germination, we compared the germination rates of the diverse *NF-Y* mutant seeds in either the presence or absence of the GA biosynthesis inhibitor paclobutrazol (PAC), respectively. Intriguingly, *nf-yc3 nf-yc4 nf-yc9* (*nf-ycT*), the combinatorial null mutant of the closest homologues *NF-YC3*, *NF-YC4* and *NF-YC9* in *Arabidopsis* NF-YC subfamily, similar to the key GA-related germination repressor mutant *rgl2* (ref. 16), showed a strong resistance to PAC, but it has no difference in germination rate with the wild-type under mock treatment (Fig. 1a,b). By contrast, there was no significant difference in germination performance between the single or double mutants of *NF-YC3*/*4*/*9* and the wild type (Supplementary Fig. 1a,b). These results suggest redundant roles of *NF-YC3*, *NF-YC4* and *NF-YC9* in repression of the GA-mediated seed germination. Consistently, the seeds of *35S:NF-YC3* and *35S:NF-YC9* exhibited lower germination rates than the wild type in the presence of a low concentration (0.5 μM) of PAC (Supplementary Fig. 1c,d). The analysis of gradient PAC concentrations further confirmed that *35S:NF-YC9* was hypersensitive, and *nf-ycT* showed reduced sensitivity to PAC, in comparison with the wild type (Fig. 1c). Given that stratification increases *Arabidopsis* seeds sensitivity to GA during germination^38^, we also examined germination phenotype of *nf-ycT* mutant in the absence of stratification. Similar to those with stratification, *nf-ycT* seeds showed significant resistance to PAC as *rgl2* under non-stratification conditions (Supplementary Fig. 2). These results indicated that NF-YC homologues negatively regulate GA-mediated seed germination.

Previous studies have shown that the GA-deficient mutant *ga1* fails to germinate, which can be sufficiently recovered by exogenous GA or loss of function of *RGL2* (refs 7, 16). Considering that the similar observations on the *nf-ycT* and *rgl2* mutants, and as a P450 enzyme inhibitor, PAC might have undesirable effect on other metabolic pathways including potentially increasing ABA levels through inhibition of ABA catabolism^39^,^40^, we thus examined whether NF-YCs could also affect *ga1* germination as RGL2. Consistent with previous report^41^, different concentrations of GA gradually increased the germination rate of *ga1*. *nf-ycT* significantly increased GA responsiveness in *ga1*, whereas overexpression of *NF-YC9* decreased that in *ga1* (Fig. 1d). Further observations showed that, besides of *nf-ycT*, the *NF-YC* double mutants also partially rescued *ga1* phenotype although the *nf-yc* single mutants had less effect on that (Supplementary Fig. 3a,b). Combined with that *NF-YC3*, *NF-YC4* and *NF-YC9* genes expressed in germinating seeds (Supplementary Fig. 4), these results support that *Arabidopsis* NF-YC3, NF-YC4 and NF-YC9 redundantly function as negative regulators in GA-mediated seed germination.

### NF-YCs interact with RGL2

**Figure 2:**
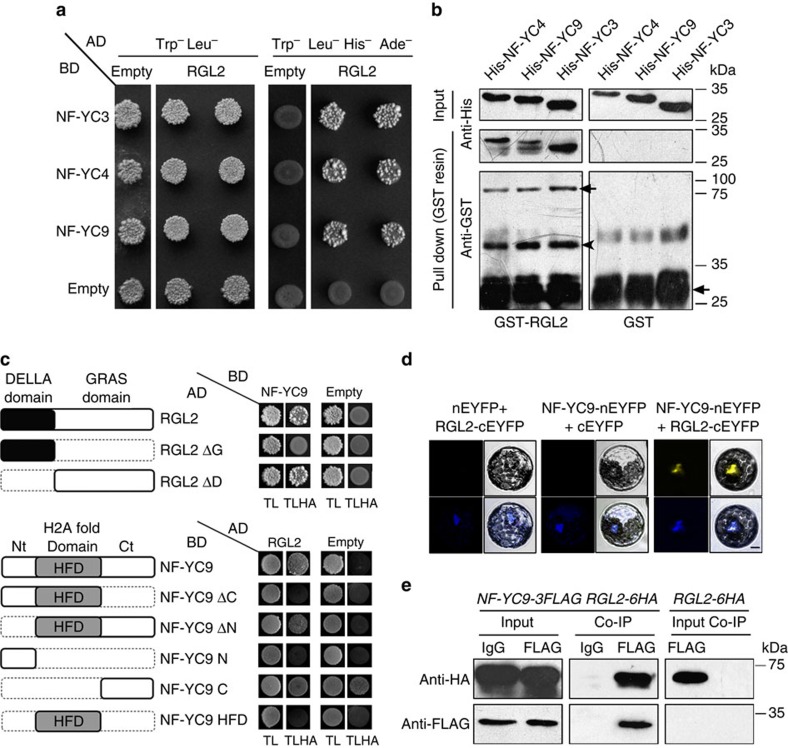
NF-YCs interact with RGL2 *in vitro* and *in vivo*. (**a**) Yeast two-hybrid assays show the interactions between RGL2 and NF-YCs. Transformed yeast cells were grown on SD/-Trp/-Leu/-His/-Ade and SD/-Trp/-Leu medium. (**b**) Pull-down assays show the direct interaction between His-NF-YCs and GST-RGL2 fusion proteins *in vitro*. His-NF-YC proteins were incubated with immobilized GST or GST-RGL2 proteins, and immunoprecipitated fractions were detected by anti-His and anti-GST antibodies, respectively. Arrows indicate the specific bands of GST-RGL2 or GST, while arrowhead indicates the nonspecific bands. (**c**) Sketches show the domains of NF-YCs and RGL2 and their various deletions. Yeast two-hybrid assays show the interactions between RGL2, NF-YCs and their derivatives. Transformed yeast cells were grown on SD/-Trp/-Leu/-His/-Ade (TLHA) and SD/-Trp/-Leu (TL) medium. (**d**) BiFC analysis of interaction between NF-YC9-nEYFP and RGL2-cEYFP in *Arabidopsis* mesophyll protoplast. DAPI staining was used as the nucleus indicator. Scale bar, 10 μm. (**e**) *In vivo* interaction of NF-YC9 and RGL2 in *Arabidopsis*. Plant nuclear extracts from PAC-treated seeds of *nf-yc9 NF-YC9:NF-YC9-3FLAG rgl2 pRGL2:RGL2-6HA* were immunoprecipitated by either anti-FLAG antibody or preimmune serum (IgG). The co-immunoprecipitated proteins were detected by anti-FLAG and anti-HA antibodies.

The analogous genetic roles of NF-YCs and RGL2 prompted us to investigate whether NF-YCs functionally associate with RGL2 to regulate seed germination. We first found that AD-RGL2 interacted with BD-NF-YC3, BD-NF-YC4 and BD-NF-YC9 in yeast, respectively (Fig. 2a). Glutathione S-transferase (GST) pull-down assays showed that each His-NF-YC was precipitated by GST-RGL2 but not by GST alone (Fig. 2b), indicating the physical interaction between RGL2 and NF-YCs *in vitro*. Because NF-YC9-3FLAG fully rescued the PAC reduced-sensitivity phenotype of *nf-yc9* and *nf-ycT* (Supplementary Fig. 5), we thus focused NF-YC9 as representative of NF-YC homologues for further investigation.

To identify whether the functional domains are required for the NF-YC and RGL2 interaction, the various truncated versions of RGL2 and NF-YC9 were used in yeast two-hybrid assays (Fig. 2c). The results showed that deletion of the GRAS domain^42^ (RGL2ΔG) prevented RGL2 binding to NF-YC9, whereas deletion of the RGL2 amino-terminal (RGL2ΔD) which excluded the entire DELLA domain did not affect the interaction between RGL2 and NF-YC9 (Fig. 2c). On the other hand, RGL2 interacted with full length of NF-YC9 and deletion of the amino-terminal (NF-YC9ΔN) but not with the HFD^26^ and the amino-terminal fragment (NF-YC9N) of NF-YC9 (Fig. 2c), indicating the carboxy-terminal fragment of NF-YC9 is necessary for interacting with RGL2 at least, although it alone revealed a self-activation in yeast. Therefore, these results suggest that the GRAS domain of RGL2 and carboxy-terminal of NF-YC9 contribute to interaction between RGL2 and NF-YC9, and may be indispensible components in potential biological function of this heterodimer.

We next performed bimolecular fluorescence complementation (BiFC) analysis to examine the interaction between NF-YC9 and RGL2 in plants. The results showed that the interaction fluorescence of NF-YC9-nEYFP with RGL2-cEYFP existed in the cell nuclei, but no YFP signal was detected in the negative control (Fig. 2d). To perform co-immunoprecipitation assay, we further created *nf-yc9 rgl2 pNF-YC9:NF-YC9-3FLAG pRGL2:RGL2-6HA* homozygous lines in which NF-YC9-3FLAG expressed at comparable levels in the germinating seeds with mock and PAC treatment, whereas RGL2-6HA was only detected in those with PAC (Supplementary Fig. 6). Both NF-YC9 and RGL2 fusion proteins retained the biological function in seeds as they are able to rescue the PAC reduced-sensitivity phenotype of mutants (Supplementary Fig. 5). The co-immunoprecipitation results of PAC-treated *nf-yc9 rgl2 pNF-YC9:NF-YC9-3FLAG pRGL2:RGL2-6HA* seeds further confirmed the *in vivo* binding of NF-YC9 to RGL2 (Fig. 2e). Taken together, these data consistently support the direct interactions between the three NF-YC homologues and RGL2 proteins.

Among DELLAs, RGL2 has been reported as the predominant repressor, and RGA, GAI and RGL1 play minor roles in GA-mediated seed germination in *Arabidopsis* L*er* ecotype^16^,^19^. Our observations confirmed that the non-germinating phenotype of *ga1* was also partially suppressed by loss of *RGA* function or completely rescued by the *rga rgl2* double mutant in *Arabidopsis* Col ecotype (Supplementary Fig. 7a). Consistent with this, like RGL2, RGA was shown to interact with NF-YC3, NF-YC4 and NF-YC9 in yeast, respectively (Supplementary Fig. 7b), which implies widespread interactions between NF-YCs and DELLAs.

### NF-YCs and RGL2 interdependently regulate seed germination

**Figure 3:**
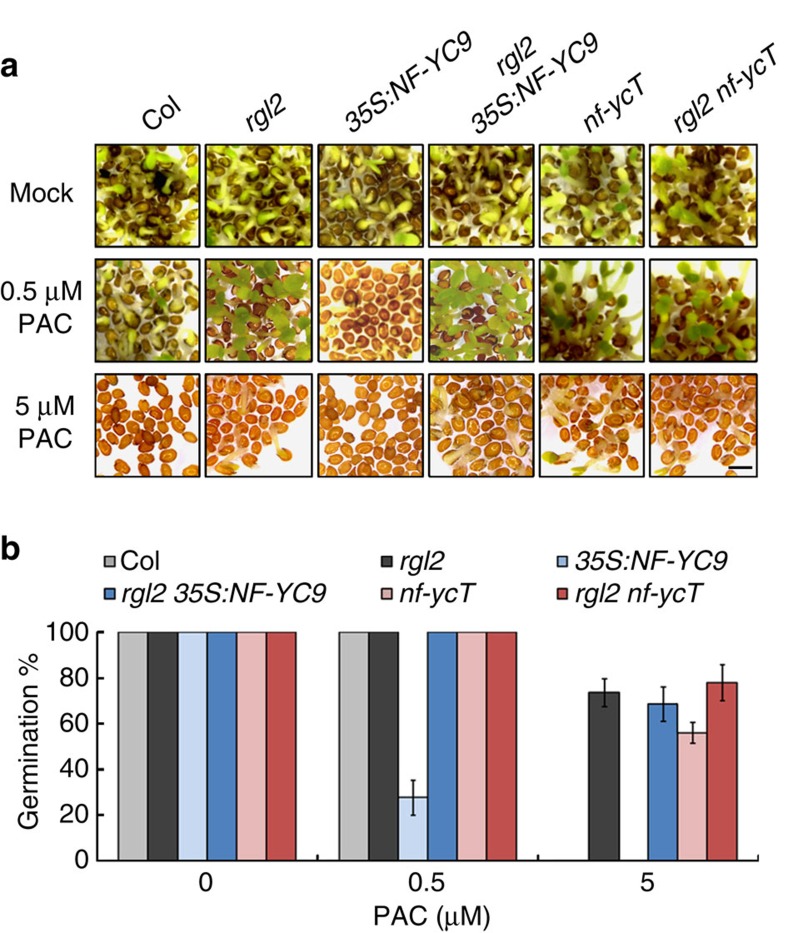
RGL2 compromises the PAC hypersensitivity of *NF-YC9* overexpression line in seed germination. (**a**) Germination phenotypes of *rgl2*, *35S:NF-YC9*, *rgl2 35S:NF-YC9*, *nf-ycT*, *rgl2 nf-ycT* and the wild-type (Col) seeds observed at 96 HAS on 1/2 MS medium containing different concentrations of PAC. Scale bar, 1 mm. (**b**) Statistic analysis of germination rate in the seeds described in **a**. Data represent mean±s.d. of at least 100 seeds.

The interaction between NF-YCs and RGL2 in plants suggests that these proteins may function together to regulate seed germination. To verify this hypothesis, we created various combinatorial genetic backgrounds of *NF-YC* and *RGL2* by intercrossing. Investigations of germination rate showed that *rgl2* fully suppressed the hypersensitivity of *35S:NF-YC9* to PAC (Fig. 3a,b). Consistently, although *35S:NF-YC9* enhanced the germination inhibition of *ga1* at low GA condition (0.01–1 μM), it did not suppress *ga1* germination at higher GA level (10 μM GA; Fig. 1d). In addition, *35S:NF-YC9* also rarely affected germination of seeds grown in normal condition (Fig. 3). Since RGL2 proteins are highly accumulated in *ga1* or under PAC treatment (Supplementary Fig. 6) and degraded in response to GA by the SCF^SLY1^ complex^23^, these observations indicate that NF-YC function on germination inhibition requires RGL2. On the contrary, similar to *rgl2*, the *nf-yc* mutants promote the germination of *ga1* or PAC-treated seeds (Fig. 1c,d, Supplementary Fig. 3a,b), supporting that the repressive role of RGL2 in germination is also dependent on NF-YCs. Observation that *rgl2 nf-ycT* had no significant difference with *rgl2* further confirmed the interdependent roles of NF-YCs and RGL2 in germination inhibition (Fig. 3).

Further analysis demonstrated that transcriptional and protein levels of NF-YC3, NF-YC4 and NF-YC9 were rarely affected by *rgl2* under mock or PAC treatment (Supplementary Fig. 8a,c). In turn, NF-YCs also had less effect on the transcription and protein accumulation of RGL2 (Supplementary Fig. 8b,c,d). These observations demonstrate that NF-YCs and RGL2 do not regulate each other in the mRNA or protein levels. Together, above data corroborate that NF-YCs and RGL2 interdependently repress seed germination via protein interactions.

### NF-YCs and RGL2 coregulate a set of downstream genes

**Figure 4:**
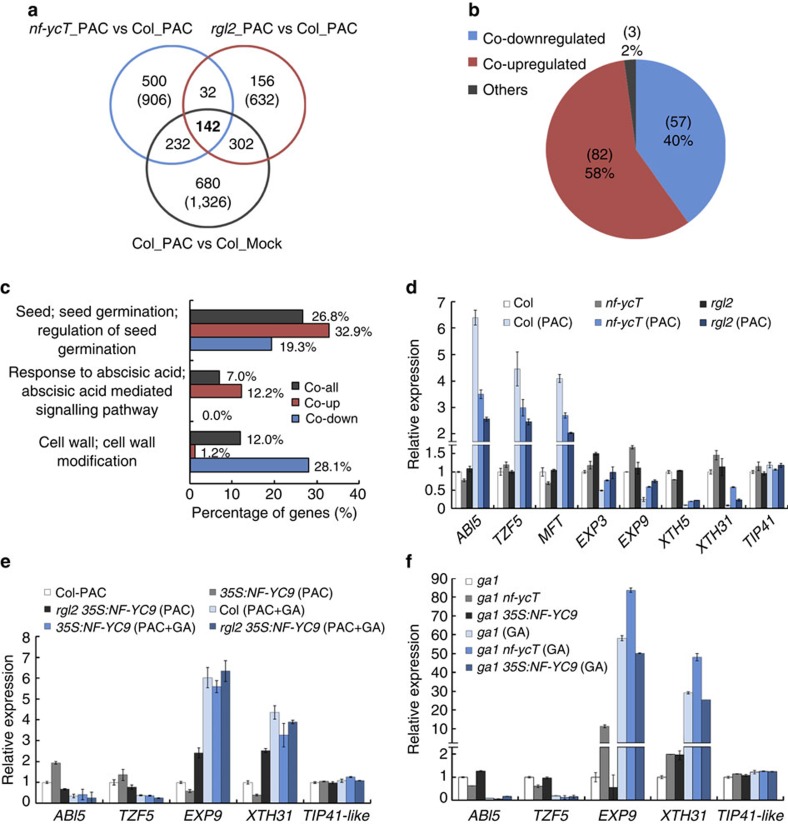
Transcriptomic analysis of regulatory gene expression profiles by NF-YCs and RGL2 in response to GA. (**a**) The Venn diagram shows the coregulated genes by GA, NF-YCs and RGL2, indicated as the overlap among the differentially expressed genes in *nf-ycT*_PAC versus Col_PAC, *rgl2*_PAC versus Col_PAC, and Col_PAC versus Col_Mock. (**b**) Percentages of the co-upregulated, the co-downregulated and the genes in the other patterns by GA, NF-YCs and RGL2. Values enclosed in parentheses indicate the numbers of genes. (**c**) Gene ontology analysis of the coregulated (co-all), the co-upregulated (co-up) and the co-downregulated (co-down) genes by GA, NF-YCs and RGL2. Numbers indicate the percentages of genes included in each GO category. (**d**) Quantitative RT–PCR analysis of the expression level of several selected cell wall-related and ABA responsive genes coregulated by GA, NF-YCs and RGL2. The wild-type (Col), *rgl2* and *nf-ycT* seeds were grown on 1/2 MS medium containing 5 μM PAC or not for 12 HAS. The relative gene expression was normalized to that of *PP2A* internal control and then calculated by comparing the value with that in Col. *TIP41-like* gene was used as a negative experimental control. Data represent mean±s.d. of three biological replicates. (**e**) Quantitative RT–PCR analysis of the expression level of the selected coregulated genes in Col, *35S:NF-YC9* and *rgl2 35S:NF-YC9* seeds grown on 1/2 MS medium containing 5 μM PAC or 5 μM PAC plus 1 μM GA for 12 HAS. The relative gene expression was normalized to that of *PP2A* internal control and then calculated by comparing the value with that in Col with PAC treatment (Col-PAC). Data represent mean±s.d. of three biological replicates. (**f**) Quantitative RT–PCR analysis of the expression level of the selected coregulated genes in *ga1*, *ga1 nf-ycT* and *ga1 35S:NF-YC9* seeds grown on 1/2 MS medium containing 1 μM GA or not for 12 HAS. The relative gene expression was normalized to that of *PP2A* internal control and then calculated by comparing the value to that in *ga1*. Data represent mean±s.d. of three biological replicates.

To understand how the NF-YC–RGL2 complex functions in repressing seed germination, a genome-wide transcriptomic analysis was carried out using germinating seeds of *rgl2*, *nf-ycT* and the wild type (Col) with PAC and Col with mock treatment (Supplementary Data 1). On the basis of the criteria of 1.5-fold cutoff for the genes with 5% false discovery rate, we first identified 1,326, 906 and 632 differentially expressed genes in Col_PAC versus Col_mock, *nf-ycT*_PAC versus Col_PAC, and *rgl2*_PAC versus Col_PAC subsets, which are referred to as PAC-, NF-YC- and RGL2-regulated genes (Fig. 4a, Supplementary Data 2). To define whether the PAC-regulated genes respond to GA, we executed a comparative analysis of the PAC-regulated genes profile with a previous microarray data in which the GA-regulated transcripts were identified from L*er* wild-type versus *ga1-3* germinating seeds^43^,^44^, and it showed a high overlap (44.6%) between these two independent data sets despite the different ecotypes and growth conditions used (Supplementary Data 2). Venn diagrams demonstrated that ∼70% (444/632) of RGL2-regulated genes and ∼41% (374/906) of NF-YC-regulated genes were also regulated by PAC mostly in an identical pattern, respectively, while ∼28% (174/632) of RGL2-regulated genes were similarly regulated by NF-YCs (Supplementary Fig. 9a), implying the high correlation of regulation between RGL2 and NF-YCs. Total 142 overlapped genes were coregulated by PAC, NF-YC and RGL2, which are considered as target genes involving in NF-YC- and RGL2-mediated seed germination (Supplementary Data 3). Strikingly, most of these coregulated genes were regulated by NF-YC, RGL2 and PAC in the same direction, and only three genes (2%) were differentially regulated (Fig. 4b, Supplementary Fig. 9b, Supplementary Data 3). Further gene ontology (GO) analysis revealed that these genes were primarily enriched in seed germination, response to hormone stimulus, cell wall modification, transferase activity, transport and other metabolic process (Supplementary Data 3). Interestingly, in the top three GO annotations, more co-upregulated genes were involved in response to ABA (12.2%), whereas lesser of them in cell wall modification (1.2%). The co-downregulated genes were remarkably enriched in cell wall-related process (28.1%) and none of them in response to ABA (0%). By contrast, the two subsets of genes were both involved in GA-mediated seed germination (32.9 and 19.3%, respectively) (Fig. 4c). These data reveal that NF-YCs and RGL2 co-target a set of common genes in response to phytohormone signals, strongly supporting the role of NF-YC-RGL2 in seed germination regulation.

Further quantitative PCR with reverse transcription (RT–PCR) analysis was performed to confirm the regulation of NF-YC–RGL2 on several selected downstream genes. Consistent with the transcriptomic analysis, PAC dramatically induced the expression of ABA responsive genes *ABI5*, *TZF5* and *MFT*, and repressed that of cell wall-related genes *EXP3*, *EXP9*, *XTH5* and *XTH31*, respectively, in the wild-type germinating seeds, whereas these PAC-triggered expression changes were compromised by *nf-yc* and *rgl2*. By contrast, these selected genes have largely comparable expression in different genetic backgrounds when grown in normal condition (Fig. 4d). Furthermore, the effect of GA on expression of NF-YC–RGL2 downstream was analysed in various genetic backgrounds. In the absence of GA (under PAC treatment or in *ga1* background), *35S:NF-YC9* promoted the expression of *ABI5*, *TZF5* and repressed that of *EXP9*, *XTH31*, respectively. However, the transcriptional regulation effect of NF-YCs on the selected genes was significantly compromised by loss of *RGL2* or GA application, similarly, the effect of RGL2 on the selected genes in *ga1* was also attenuated by loss of *NF-YCs* or GA application (Fig. 4e,f). These results confirm that NF-YC–RGL2 differentially regulates two subsets of genes that are involved in ABA response and GA-mediated cell wall modification, respectively, to repress seed germination. Meanwhile, it is also intriguing how this complex functions on activation and repression of its downstream. Interestingly, the chromatin immunoprecipitation (ChIP) assay showed that, rather than the direct transcriptional repression on the cell wall-related genes, NF-YC–RGL2 module might directly target ABA responsive gene *ABI5* for transcriptional activation (Supplementary Fig. 10).

### NF-YC–RGL2 activates *ABI5* by recognizing CCAAT elements

**Figure 5:**
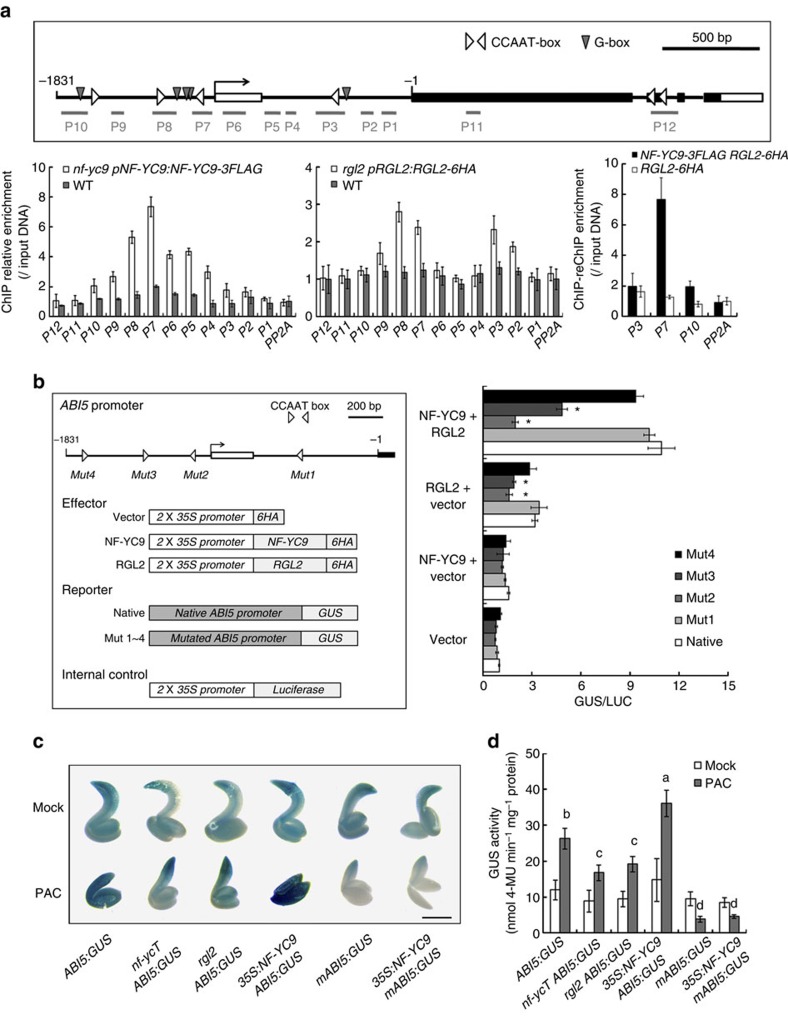
NF-YCs and RGL2 synergistically regulate *ABI5* expression by binding to the *ABI5* promoter. (**a**) ChIP and ChIP-reChIP analyses of NF-YC9 and RGL2 binding to CCAAT-box containing region in *ABI5* genes upon precipitation with anti-FLAG or/and anti-HA antibodies in the WT (wild-type, Col-0), *nf-yc9 pNF-YC9:NF-Y9-3FLAG*, *rgl2 pRGL2:RGL2-6HA* (*RGL2-6HA*) and *rgl2 nf-yc9 pNF-YC9:NF-YC9-3FLAG pRGL2:RGL2-6HA* (*NF-YC9-3FLAG RGL2-6HA*) lines. The seeds were grown on 1/2 MS medium containing 5 μM PAC for 12 HAS and harvested for further test. Relative enrichment fold was calculated by normalizing the amount of a target DNA fragment against that of a genomic fragment of a reference gene *TUB8*, and then against the respective input DNA samples. The enrichment of a *PP2A* genomic fragment was used as the negative control (the same below). Data represent mean±s.d. of biological triplicates. (**b**) Transient expression assays of *ABI5* promoter activity modulated by NF-YC9 and RGL2 in *Arabidopsis* mesophyll protoplasts. Various constructs used in transient expression assays are shown in the left panel. Either *ABI5:GUS* (Native) or four *mABI5:GUS* (Mut1∼4) were co-transformed with effectors or the empty vector (Vector) into Col mesophyll protoplasts. Relative GUS activity (GUS/Luciferase) that indicates the level of *ABI5* expression activated by various effectors is shown in the right panel. Data represent mean±s.d. of three biological replicates. Asterisks indicate significant changes of samples when compared with the relevant native sample (Student's *t*-test, *P*<0.05). (**c**) Representative GUS staining of mock (upper panel) and PAC treated (lower panel) seeds harbouring *ABI5:GUS* or its mutated version in various genetic backgrounds. The seeds were grown on 1/2 MS either containing 5 μM PAC or mock for 48 HAS. Scale bar, 0.5 mm. (**d**) Quantitative analysis of GUS activity in the native and mutated *ABI5:GUS* seeds shown in **c**. Data represent mean±s.d. from at least 100 seeds of each genotype. Statistically significant differences are indicated by different lower-case letters (Student's *t*-test, *P*<0.05).

The binding of NF-YC–RGL2 to chromatin provoked us to speculate whether this complex serves as a transcriptional activator to directly regulate the *ABI5* gene. Because NF-Y was reported to specifically bind to the CCAAT-box in promoter of target genes^26^,^45^, we analysed the *ABI5* genomic DNA and chose 12 fragments (P1 to P12), which covered all six CCAAT-boxes of the *ABI5* region, for next examination (Fig. 5a). ChIP analyses of PAC*-*treated *nf-yc9 pNF-YC9:NF-YC9-3FLAG* and *rgl2 pRGL2:RGL2-6HA* seeds revealed that both NF-YC9 and RGL2 were associated with the genomic region near the adjacent fragments P7 and P8 with the highest enrichments (Fig. 5a). ChIP–reChIP analysis further verified that NF-YC9 and RGL2 co-localized to the same region of *ABI5* (Fig. 5a). Since P7 and P8 fragments contained two CCAAT elements (designated as CCAAT-2 and CCAAT-3, respectively), to examine whether these elements are involved in the NF-YC–RGL2 regulation on *ABI5* expression, we performed transient expression assays using ∼1.8 kb fragment of native or various mutated *ABI5* promoters fused to the β-glucuronidase (*GUS*) reporter gene. The effector constructs of *35S:NF-YC9* and *35S:RGL2* were individually or together transfected with reporters into *Arabidopsis* mesophyll protoplasts (Fig. 5b). Addition of RGL2 or NF-YC9 activated the expression of *ABI5*. Notably, in comparison with that expressing RGL2 alone, the higher GUS activity was detected when co-expressing NF-YC9 and RGL2 (Fig. 5b). However, when site-specific mutations (CCAAT to ACATA) were introduced into the CCAAT elements in *ABI5* promoter, the expression of *ABI5* was strikingly impaired by disruption of the CCAAT-2 or CCAAT-3 (Mut2 or Mut3) but not by Mut1 or Mut4, either each or both NF-YC9 and RGL2 existed (Fig. 5b). These results indicate that the CCAAT elements located at P7 and P8 are essential for NF-YC–RGL2-mediated activation of *ABI5*. In addition, other DELLA proteins also contribute to *ABI5* expression activation together with NF-YC9 in a variable extent (Supplementary Fig. 11). Since the different circumstances between cells of protoplasts and germinating seeds, the biological roles of these DELLAs on *ABI5* in plants still need to be further determinated.

Because no DNA binding domain is identified in DELLA proteins, to make clear how RGL2 and NF-YC9 recognize *ABI5* promoter, we performed protein–DNA affinity pull-down assay in which a heterotrimeric NF-Y, composed of NF-YC9 and two NF-Y subunits core domains from yeast (NF-YA core and NF-YB core), was used for CCAAT element binding as previously reported^46^. Interestingly, RGL2 indirectly associated with the *ABI5* promoter fragment DNA containing CCAAT-2/3 (*ABI5-2,3*), but not with the mutated *ABI5-2,3* or *ABI5* promoter fragment containing CCAAT-4 (*ABI5-4*), via interacting with NF-YA core/NF-YB core/NF-YC9 complex (Supplementary Fig. 12). However, the DNA affinity of RGL2-NF-YC9 was not detected *in vitro* (Supplementary Fig. 12), which is consistent with the previous reports that NF-YC subunit has no DNA binding ability^26^. These results indicate that RGL2, not itself, but via NF-Y complex, recognizes *ABI5* promoter region.

To further examine whether NF-YC–RGL2 regulates *ABI5* expression through the CCAAT elements during seed germination, we created *ABI5:GUS* transgenic plants and two mutated lines containing the Mut2 or Mut3 version of CCAAT elements. Among 11 transformants harbouring Mut2 (*mABI5:GUS*) with PAC treatment, 9 displayed significantly reduced GUS staining in comparison with *ABI5:GUS* seeds (data not shown). The similar results were observed in the transgenic lines harbouring Mut3 (*m3-ABI5:GUS*) (Supplementary Fig. 13a,b). These observations verified that CCAAT-2 and CCAAT-3 are critical for GA-mediated *ABI5* expression during seed germination. Furthermore, with PAC treatment, the staining of *ABI5:GUS* seeds was remarkably weaker in both *nf-ycT* and *rgl2* than that in the wild-type background, whereas there was no significant difference of GUS staining among these germinating seeds with mock treatment (Fig. 5c,d). As expected, *ABI5:GUS* but not *mABI5:GUS* seeds displayed an increased GUS staining in *35S:NF-YC9* compared with the wild-type background (Fig. 5c,d). Thus, these findings strongly support the idea that NF-YC–RGL2 module activates *ABI5* expression via binding to the specific CCAAT elements. It was noted that, although PAC resulted in increased GUS staining in the *ABI5:GUS* seeds, but it had an opposite effect on the *mABI5:GUS* seeds probably owing to other unknown regulations caused by the disruption of CCAAT element.

### *ABI5* is epistatic to *NF-YCs* and *RGL2*

**Figure 6:**
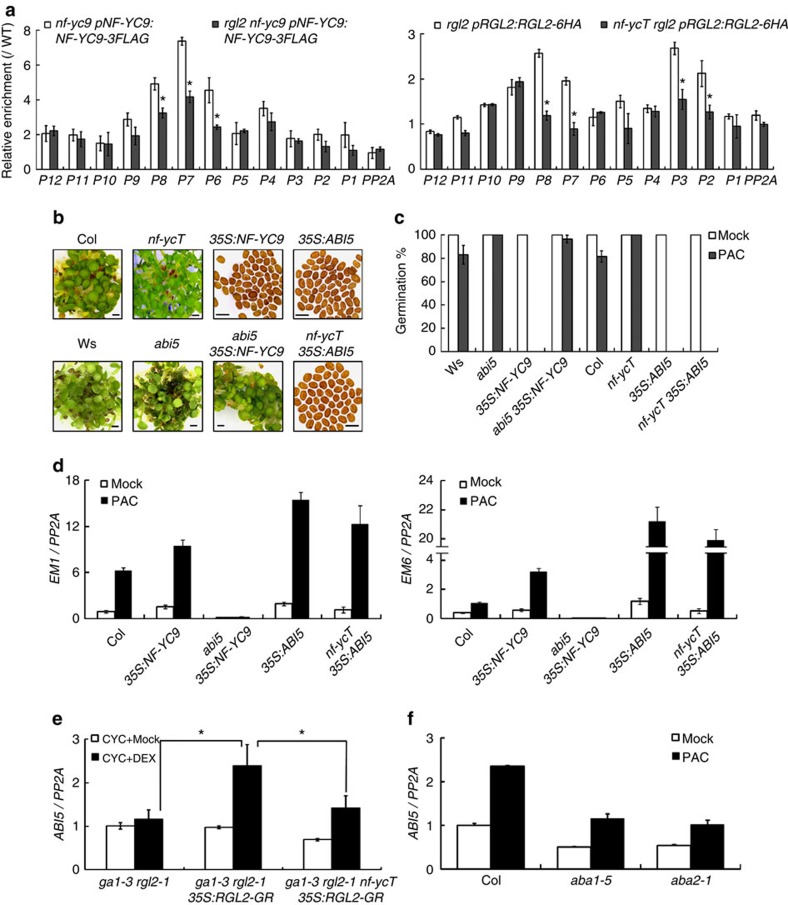
ABI5 is required for NF-YC–RGL2-mediated seed germination. (**a**) ChIP analysis of NF-YC9 or RGL2 binding to CCAAT-box containing regions of *ABI5* promoter upon precipitation with anti-FLAG or anti-HA antibodies in seeds under various genetic backgrounds. The seeds were grown on 1/2 MS medium containing 5 μM PAC for 12 HAS and harvested for ChIP assay. Data represent mean±s.d. of triplicates. Asterisks indicate significant changes in ChIP-enrichment fold between crossed lines and relevant parent lines (Student's *t*-test, *P*<0.05). (**b**) Germination phenotypes of seeds in various genetic backgrounds observed at 120 HAS on 1/2 MS medium containing 1 μM PAC. (**c**) Statistic analysis of germination rate in the seeds described in **b**. Germination rate in all the seeds with mock treatment is 100%. Data represent mean±s.d. of at least 100 seeds. Scale bar, 1 mm. (**d**) Quantitative RT–PCR analysis of *EM1* and *EM6* expression in seeds in various genetic backgrounds grown on 1/2 MS medium containing 5 μM PAC or not for 12 HAS. (**e**) Quantitative RT–PCR analysis of *ABI5* expression in *ga1-3 rgl2-1*, *ga1-3 rgl2-1 35S:RGL2-GR* and *ga1-3 rgl2-1 nf-ycT 35S:RGL2-GR* seeds grown on 1/2 MS medium containing 10 μM cyclohexanone (CYC) or 10 μM CYC plus 10 μM dexamethasone (DEX) for 4 h. Data represent mean±s.d. of three replicates. Asterisks indicate significant changes between the selected samples (Student's *t*-test, *P*<0.05). (**f**) Quantitative RT–PCR analysis of *ABI5* expression in *aba1-5*, *aba2-1* and the wild-type seeds grown on 1/2 MS medium containing 5 μM PAC or not for 12 HAS. Data represent mean±s.d. of three replicates.

Because NF-YCs and RGL2 interact to directly regulate *ABI5* expression, we wondered whether NF-YCs and RGL2 are cooperative and interdependent on such transcriptional regulation. ChIP analyses of *rgl2 nf-yc9 pNF-YC9:NF-YC9-3FLAG* and *nf-ycT rgl2 pRGL2:RGL2-6HA* showed that the absence of RGL2 significantly impaired the affinity of NF-YC9 to P7/8 fragments in *ABI5* promoter. In turn, binding of RGL2 to the same locus was attenuated by *nf-ycT* (Fig. 6a). Furthermore, GA application also weakened the DNA binding of NF-YC9 in PAC-treated seeds (Supplementary Fig. 14). The results suggest that NF-YCs and RGL2 cooperatively bind to the CCAAT elements to regulate *ABI5* transcription.

ABI5 functions as the central ABA signalling component to repress seed germination, which is epistatic to *RGL2* (ref. 23). To investigate the genetic role of *NF-YCs* in ABI5-mediated inhibition of seed germination, we created *abi5 35S:NF-YC9* combinatorial line and found that loss of *ABI5* strikingly suppressed the hypersensitivity of *35S:NF-YC9* to PAC in germinating seeds (Fig. 6b,c). Consistent with this, the expression of two ABI5 target genes *EM1* and *EM6* was decreased in *abi5 35S:NF-YC9* compared with *35S:NF-YC9* seeds (Fig. 6d). In turn, overexpression of *ABI5* remarkably rescued the PAC-reduced sensitivity of *nf-ycT* (Fig. 6b,c), and *EM1* and *EM6* genes expressed at comparable levels in *35S:ABI5* and *nf-ycT 35S:ABI5* seeds (Fig. 6d). These results, together with previous report^23^, suggest that *ABI5* act epistatic to NF-YCs and RGL2 during seed germination. We next examined the *ABI5* expression in response to immediate RGL2 activity using a steroid-inducible RGL2 (RGL2-GR) in *ga1-3 rgl2-1* background. In contrast to no change in *ga1-3 rgl2-1*, *ABI5* expression was rapidly induced by dexamethasone combined with cycloheximide in *ga1-3 rgl2-1 35S:RGL2-GR* without *de novo* protein synthesis, whereas it was compromised in *ga1-3 rgl2-1 nf-ycT 35S:RGL2-GR* (Fig. 6e), providing a further molecular evidence to support genetic relationship between NF-YC–RGL2 and *ABI5*.

Previous studies showed that GA promotes seed germination by triggering RGL2 degradation, and RGL2 indirectly regulates *ABI5* expression through stimulating ABA biosynthesis, thus inhibiting germination^23^,^47^,^48^. Here, our observations support a direct regulation of RGL2 on *ABI5*. To determine whether such regulation is ABA dependent or not, we examined the expression of *ABI5* in ABA synthesis defective mutants *aba1-5* and *aba2-1*. Interestingly, the *ABI5* expression was still induced, even though with lesser elevated extent than the wild-type, in PAC-treated *aba1* and *aba2* seeds compared with mock-treated (Fig. 6f), implying *ABI5* might be regulated by GA or RGL2 regardless of ABA biosynthesis.

### NF-YC–RGL2 functions in ABA-mediated germination inhibition

**Figure 7:**
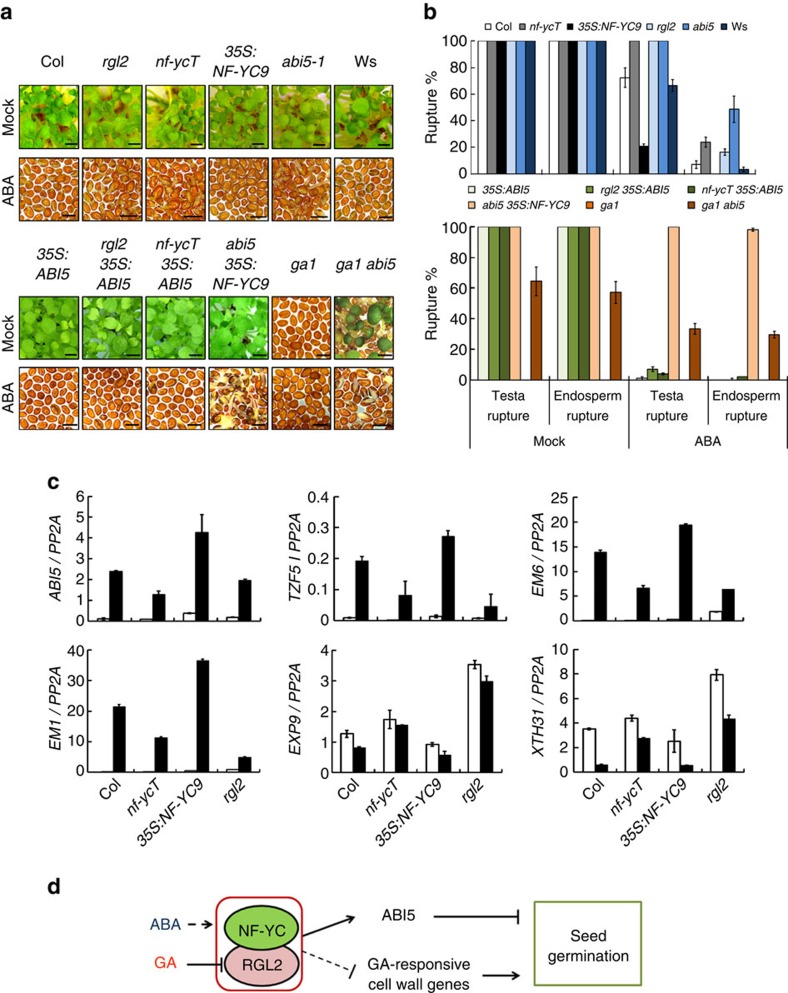
NF-YCs and RGL2 are involved in the ABA-mediated inhibition of germination. (**a**) Germination phenotypes of *rgl2*, *nf-ycT*, *abi5*, *35S:NF-YC9*, *35S:ABI5*, the wild-type (Col and Ws) seeds and their combinatorial lines observed at 120 HAS on 1/2 MS medium containing 2 μM ABA or not (mock). Scale bar, 1 mm (**b**) Statistic analysis of the testa and endosperm rupture rate in germinating seeds described in **a**. Data represent mean±s.d. of at least 100 seeds. (**c**) Quantitative RT–PCR analysis of several representative genes expression in *nf-ycT*, *35S:NF-YC9*, *rgl2* and the wild-type seeds grown on 1/2 MS medium containing 2 μM ABA or not for 12 HAS. Data represent mean±s.d. of three replicates. (**d**) A model of NF-YC-RGL2-mediated seed germination by integrating GA and ABA signalling. Solid lines indicate the direct or definite regulation; dotted lines indicate the indirect or undetermined regulation.

Germination process contains the rupture of the testa before the endosperm rupture. ABA suppresses seed germination mainly through inhibition of endosperm rupture rather than that of testa rupture, whereas RGL2 accumulation prevents both testa and endosperm rupture under GA deficiency^10^,^23^. We here investigated the detailed role of NF-YCs in testa and endosperm rupture. Under normal condition, the germination rates of all examined seeds were 100% at 120 HAS (Fig. 7a,b). In the presence of ABA, compared with the wild-type, *nf-ycT*, *rgl2* and *abi5* showed higher testa and endosperm rupture rates, but the *35S:NF-YC9* seeds had the lower rate on those (Fig. 7a,b). Furthermore, *35S:ABI5* dramatically attenuated or abolished the ABA insensitivity of *nf-ycT* and *rgl2*, while *abi5 35S:NF-YC9* still remained high testa and endosperm rupture rates as *abi5*. In addition, loss of *ABI5* function also significantly rescued non-germinating phenotype of *ga1* under either ABA or mock treatment (Fig. 7a,b). The results indicate that, similar to RGL2, NF-YCs repress both testa and endosperm rupture via *ABI5* in ABA-mediated seed germination.

To further determine the regulatory effect of NF-YC–RGL2 module on ABA-mediated seed germination, we examined the transcriptional level of several representative NF-YC–RGL2 coregulated genes in different seeds under ABA treatment. As expected, all selected genes in the ABA-treated seeds of *nf-ycT*, *rgl2* and *35S:NF-YC9* were expressed in similar regulatory patterns with those under PAC treatment (Fig. 7c, Fig. 4d,e, Supplementary Fig. 15). These results collectively support the conclusion that NF-YC–RGL2 module integrates ABA and GA signalling to regulate seed germination.

## Discussion

Numerous genetic and physiological studies have documented the antagonistic roles of GA and ABA, which are essential for seeds to determine whether germination starts or not. DELLA protein RGL2, the main GA signalling repressor in germination, serves as a central modulator in such process^16^,^19^,^20^. GA-triggered degradation of DELLAs by ubiquitin–proteasome pathway or repression of DELLAs by nonproteolytic GA signalling promotes normal seeds germination^18^,^47^,^48^. Here, we demonstrate that three *Arabidopsis* NF-YC homologues interact with RGL2 protein to interdependently regulate a set of genes involved in GA-related cell wall modification and ABA response, especially *ABI5*, the gene encoding a core component of ABA signalling, thus, control seed germination (Fig. 7d). In imbibing seeds, bioactive GA is produced to decrease RGL2 accumulation, thus mediating ABI5-regulated ABA signalling and accelerating germination process. These results illustrate a hypothetic regulatory model of phytohormones crosstalk and reveal a direct molecular link of NF-YC–RGL2–ABI5 that integrates GA and ABA signalling to precisely regulate seed germination, providing new insights into understanding on how DELLAs mediate the antagonism between GA and ABA via a direct signalling modulation.

Consistent with antagonistic roles of GA and ABA in germination, GA synthesis promptly ascends, while ABA content decreases, in imbibed seeds^4^,^5^. Furthermore, it has been showed that the ABA synthesis deficient mutant *aba2* seeds have higher endogenous GA levels^49^, and in turn, the ABA synthesis is enhanced in the GA-deficient mutant *ga1-3* (ref. 41). ABI5 plays a vital role in repressing the germination of nondormant seeds, and its transcriptional expression and protein activity respond to changes in ABA and GA levels. The studies have suggested that RGL2 stimulates endogenous ABA synthesis probably via XERICO, a RING-H2 factor promoting ABA accumulation in an unknown manner, thus activating *ABI5* expression^23^,^25^. However, we here reveal a direct regulation of RGL2 in *ABI5* transcription through interacting with NF-YCs. This is further corroborated by the observations that PAC induces the expression of *ABI5* even in the absence of ABA (*aba1* and *aba2* background), and immediate upregulation of *ABI5* by the inducible RGL2 does not need *de novo* protein synthesis (Fig. 6e,f), suggesting that RGL2 is able to directly regulate *ABI5* gene in an ABA-independent manner. It is also noteworthy that the PAC*-*induced *ABI5* expressions are lower in *aba1-5* and *aba2-1* than that in the wild-type (Fig. 6f). This is probably due to the decreased mRNA and protein level of RGL2 in ABA-deficient mutants^23^. Considering that the stability and activity of ABI5 protein are mostly dependent on ABA levels^23^, and changes of ABA or ABI5 level affect the sensitivity of germinating seeds to PAC^13^ (Fig. 6b,c), ABA may act as an important modulator in fine-tuning the regulation of NF-YC–RGL2–ABI5 hierarchical cascade on seed germination.

The transcriptomic analysis reveals that the RGL2–NF-YC modulates two subsets of different downstreams including ABA responsive and cell wall-related genes in germination. It is noteworthy that in addition to the main effect in blocking GA biosynthesis, PAC used in this analysis might cause an undesirable increase of ABA levels by interfering in ABA catabolism^39^. A comparative analysis of the PAC-regulated genes with a previously identified GA-regulated expression profile^43^ exhibited high overlapping between these two independent data sets, together with the expression analysis of the selected genes, supporting that the majority of PAC-regulated genes is responsive to GA. *TZF4/SOM* and *TZF5*, encoding two functional CCCH zinc finger proteins presented in co-upregulated genes profile, repress seed germination by controlling GA and ABA responsive genes expression^50^,^51^. MFT, another co-upregulated gene product, is involved in seed germination regulation through a negative feedback loop modulating ABA signalling pathway^52^. We hence speculate that NF-YC–RGL2 might act as a key node to induce ABA responsive gene expression and to repress GA-related cell wall genes expression partially via activating a number of transcriptional regulators such as ABI5, TZF4/SOM, TZF5 and MFT (Fig. 4, Supplementary Data 3). The α-amylase gene encodes starch hydrolase and acts as a classic downstream gene under the antagonistic regulation between GA and ABA in cereal seeds germination^53^. In the barley aleurone, *GAMYB*, encoding a well-known GA-related transcription factor that induces the expression of α-amylase gene, is promoted by GA-triggered degradation of DELLA protein SLN1 and repressed by the ABA-induced protein kinase PKABA1 (refs 53, 54). Our transcriptomic analysis and ChIP assays did not detect the direct regulation by DELLA-NF-YC module in these genes. How α-amylase encoding *AtAMYs* and *GAMYB* are regulated by GA and ABA signalling in *Arabidopsis* germinating seeds, and whether the DELLA-NF-YC module works in cereal plants, remain to be investigated in future.

As transcriptional regulators, DELLA proteins exert their function by interfering with other transcription factors rather than directly binding to their target genes^55^,^56^,^57^. However, the increasing evidences revealed that DELLAs also likely activate or repress downstream genes expression through directly targeting their promoters^25^,^44^,^58^,^59^,^60^. Our observation of *ABI5* activation by NF-YC–RGL2 further confirm this, supporting the dual role of DELLA presented in ‘the targeting model' and ‘relief of repression model' as described previously^57^,^58^. NF-YC associates with NF-YA and NF-YB subunits by the HFD domain for recognition of CCAAT element in eukaryotes^26^. Recent studies also showed the CCAAT binding of plant NF-Y heterotrimer by several combinations of NF-Y subunits *in vitro* or *in vivo*^31^,^33^,^45^,^61^. In addition, canonical CCAAT boxes were identified as important repressive transcription regulatory elements in promoters of rice *GAMYB* and *RPBF*, the genes involved in GA regulation of expression during germination of rice seeds^62^. In our observations, RGL2 interacts with the non-HFD C terminus of NF-YCs to co-locate at the CCAAT elements in *ABI5*, implying that specific NF-Y complexes might function with RGL2 together in control of seed germination. Further protein–DNA affinity pull-down assay confirms the direct binding of NF-Y–RGL2 to the *ABI5* promoter, and that RGL2 indirectly recognizes CCAAT elements via NF-Y complex. Notably, there are three G-box elements (CACGTG) contained between the two functional CCAAT sites (Fig. 5a). These G-box are bound by ABI5 itself in yeast^63^. Hence, it raises concern that RGL2 and NF-YCs probably play a role in mediating *ABI5* self-regulation.

Previous studies reported that NF-YC3, NF-YC4 and NF-YC9 redundantly function in cooperation with GA response to promote flowering, which is in turn repressed by DELLAs via protein interaction^30^,^36^. However, our results indicate that these NF-YCs together with DELLAs oppose GA response for seed germination inhibition. Because NF-Y functions as the important regulators that widely mediate plant development and environmental responses^27^, this opposite role of NF-YCs between germination and flowering processes might be caused by the spatio-temporal regulation of NF-Y complexes consisted of diverse NF-YA/B/C on different sets of target genes in various development stages. In addition, DELLAs also have distinct functions other than overlapping roles in plant development. For instance, RGL2 functions as the central repressor in GA-mediated and light-dependent germination, while RGA and GAI synergistically repress plant vegetative growth^15^,^20^,^23^. Hence, it is worthy to identify different combinations of DELLA-NF-Y(C) that function in different biological processes, such as cell expansion, tissue development and stress responses.

Taken together, we reveal a key regulatory module NF-YC–RGL2 by which GA directly intervenes in ABA signalling, and thus regulates seed germination. These findings provide novel insights into mechanism of antagonism between GA and ABA during plant development.

## Methods

### Plant materials and growth conditions

All *Arabidopsis* plants used in this study are in Col background except for *ga1-3 rgl2-1* and *ga1-3 rgl2-1 35S:RGL2-GR* in L*er* background, and *abi5-1* in Ws background. The *nf-yc3-2* (GK-051E10), *nf-yc4-1* (SALK_032163), *nf-yc9-1* (SALK_058903), *ga1* (SALK_109115), *rgl2* (SALK_124231) and *rga-28* (SALK_089146) seeds were obtained from the ‘The Arabidopsis Information Resource (TAIR, http://www.arabidopsis.org/). Transgenic lines of *35S:NF-YC3-6HA*, *35S:NF-YC9-6HA* and *nf-yc9-1 pNF-Y9:NF-YC9-3FLAG* and the mutants of *aba1-5* and *aba2-1* (refs 13, 36). Each transgenic line used to compare under different genetic backgrounds in this study is the same line which was introduced into various mutants by crossing. The seeds used for germination comparison were harvested in the same batch of plants grown at 22 °C under long days (16 h light/8 h dark). Dry seeds were obtained and stored in a dry condition (25% humidity, 25 °C) for at least 4 weeks of after-ripening before performing the germination test.

Genes referenced in this article can be found in the *Arabidopsis* Genome Initiative database under the following accession numbers: *NF-YC3* (AT1G54830), *NF-YC4* (AT5G63470), *NF-YC9* (AT1G08970), *RGL2* (AT3G03450), *RGA* (AT2G01570), *RGL1* (AT1G66350), *GAI* (AT1G14920), *RGL3* (AT5G17490), *ABI5* (AT2G36270), *TZF5* (AT5G44260), *MFT* (AT1G18100), *EM1* (AT3G51810), *EM6* (AT2G40170), *XTH5* (AT5G13870), *XTH31* (AT3G44990), *EXP3* (AT2G37640), *EXP9* (AT5G02260), *PP2A* (AT1G13320), *TIP41-like* (AT4G34270) and *TUB8* (AT5G23860).

### Seed germination assay

The after-ripened seeds were sterilized and washed with 75% (v/v) ethanol with 0.5% (v/v) Triton X-100 (Sigma-Aldrich) for 1 min, and washed twice with absolute ethanol, then were plated on sterile filter paper for air drying. Subsequently, the sterilized seeds were sown on half-strength MS medium (0.025% MES, pH 5.7) containing 0.8% (w/v) Bacto Agar (Difco/BD) supplemented with 0.01% (w/v) ethanol (Mock), PAC (Sigma-Aldrich), ABA (Sigma-Aldrich), GA_3_ (Sigma-Aldrich) or DEX (Sigma-Aldrich) upon the experiment requirement. All the plates were kept at 4 °C in darkness for 3 days for stratification and then transferred to an illumination incubator at 22 °C with 16 h light/8 h dark condition for further analysis. At least 100 seeds for each genotype were used in three biological replicates. The germination event was defined as the first sign of radicle emergence and recorded at different time points until 120 h of incubation.

### Plasmid construction and plant transformation

For the *pRGL2:RGL2-6HA* construct, an ∼3.7 kb genomic fragment of *RGL2* without stop codon was amplified and cloned into the pHY105-6HA binary vector^57^. To construct *35S:RGL2-6HA*, the CDS encoding RGL2 was amplified and cloned into pGreen-35S-6HA. For the *ABI5:GUS* construct, ∼1.8 kb promoter of *ABI5* was cloned into the pHY107 vector harbouring the *GUS* reporter^57^. The primers used for plasmid construction are listed in Supplementary Table 1. Transgenic plants harbouring *pNF-YC9:NF-YC9-3FLAG* were selected on 1/2 MS medium supplemented with gentamicin, while other transgenic plants were selected by basta.

### Yeast two-hybrid assay

The coding regions of *NF-YC3*, *NF-YC4*, *NF-YC9*, *RGL2* and *RGA* or truncated versions of *NF-YC9* and *RGL2* were amplified and cloned into pGBKT7 and pGADT7 (Clontech), respectively. The primers used are listed in Supplementary Table 1. Yeast two-hybrid assays were performed using the Yeastmaker Yeast Transformation System 2 (Clontech). Yeast AH109 cells were co-transformed with the specific bait and prey constructs. All yeast transformants were grown on SD/-Trp/-Leu or SD/-Trp/-Leu/-His/-Ade medium for selection or interaction test.

### BiFC analysis

The coding regions of *NF-YC9* and *RGL2* were cloned into the serial pGreen binary vectors containing C- or N-terminal fusions of EYFP to generate *35S:NF-YC9-nEYFP* and *35S:RGL2-cEYFP*. Plasmids were co-transformed into *Arabidopsis* mesophyll protoplasts by the PEG-mediated transient transformation^64^, and then cultured for 12 h and observed for BiFC analysis using a confocal laser scanning microscope (LSM 510 META, Zeiss).

### *In vitro* pull-down assay

The coding regions of *NF-YC3*, *NF-YC4*, *NF-YC9* and *RGL2* were cloned into the pQE30 (QIAGEN) and pGEX-4T-1 (Pharmacia) vectors to produce His-NF-YC3, His-NF-YC4, His-NF-YC9 and GST-RGL2 proteins, respectively. Primers used for constructions are listed in Supplementary Table 1. GST and His fusion recombinant proteins were induced by IPTG and expressed in *E. coli* Rosetta (DE3, Novagen). The soluble His and GST fusion proteins were purified using Ni-NTA agarose beads (30210, QIAGEN) or Glutathione Sepharose Beads (17-0756-01, Amersham Biosciences) according to the manufacturers' instruction. For pull-down assays, 2 μg of His-NF-YCs were incubated in the binding buffer (50 mM Tris-HCl, pH 8.0, 100 mM NaCl and 1 mM EDTA) with immobilized GST or GST fusion protein at 4 °C for 2 h. After washing with binding buffer, proteins retained on the beads were subsequently resolved by SDS loading buffer and then run SDS–PAGE to detect with anti-His (AbM59012-18-PU, BGI) at a dilution of 1:5,000 or anti-GST antibody (AB101-02, Tiangen) at a dilution of 1:2,000. Uncropped scans of western blot results are shown in Supplementary Fig. 16.

### Co-immunoprecipitation assay

The 5 μM PAC-treated *nf-yc9 rgl2 pNF-YC9:NF-YC9-3FLAG pRGL2:RGL2-6HA* seeds were kept under light for 12 h. Total proteins were extracted with extraction buffer (50 mM Hepes, pH 7.5, 150 mM NaCl, 5 mM DTT, 1% Triton X-100), and were incubated with Protein G PLUS/Protein A-Agarose Suspension (IP10, CALBIOCHEN) plus either anti-FLAG antibody (F3165, Sigma) or preimmune serum (IgG) in the co-immunoprecipitation buffer (50 mM Hepes, pH 7.5, 150 mM KCl, 10 μM ZnSO_4_, 5 mM MgCl_2_, 1% Triton X-100) at 4 °C for 2 h. After being washed by co-immunoprecipitation buffer three times, the proteins bound to beads were resolved by SDS–PAGE and detected by anti-FLAG (F3165, Sigma) at a dilution of 1:10,000 or anti-HA antibody (sc-7392, Santa Cruz) at a dilution of 1:2,000. Uncropped scans of western blot results are shown in Supplementary Fig. 16.

### RNA-seq analysis

The after-ripened seeds (4 weeks) harvested in a same batch were grown on half-strength MS medium (0.025% MES, pH 5.7) containing 5 μM PAC under light for 12 h. Total RNA was extracted from harvested seeds by Plant RNA Kit (R6827, Omega) and sent to BGI for RNA-seq analysis. The used RNA samples have been strictly detected upon the RNA sequencing standard and the libraries constructed using Ultra RNA sample preparation kit (Illumina) reached high quality before RNA-sequencing. Sequencing was performed using an Illumina HiSeq2000 according to the standard protocol. Total RNA-Seq reads were mapped to the *Arabidopsis* TAIR10 genome. The differentially expressed genes were identified by the program Cuffdiff with the criteria set as fold change >1.5 and FDR-adjusted *P* values <0.05. Three valid biological replicates were used for the transcriptomic analysis. The gene expression patterns were graphically represented in a heat map by cluster analysis tool in Heml software^65^. GO analysis was performed by the GO Annotation of TAIR^66^.

### Gene expression analysis

The treatment of seeds was performed upon various experiments. Total RNA was extracted using the Plant RNA Kit (Omega) and reverse transcribed using the M-MLV reverse transcriptase (Promega). Quantitative RT–PCR was performed in triplicates on Roche LightCycler480 real-time system with the SYBR Premix ExTaq Mix (DRR041A, TaKaRa) following the manufacturer's instruction. The relative expression level was normalized to that of *PP2A* internal control. The primers used for gene expression analysis are listed in Supplementary Table 1.

### ChIP and ChIP–reChIP assays

To perform ChIP assays, the *nf-yc9 pNF-YC9:NF-YC9-3FLAG, rgl2 pRGL2:RGL2-6HA* and the Col wild-type seeds were incubated with mock, 5 μM PAC or 5 μM PAC plus 1 μM GA for 12 h and harvested for fixation. Chromatins were isolated and sonicated to generate DNA fragment with an average size around 250–500 bp. The solubilized chromatins were immunoprecipitated by Protein G PLUS agarose (16–201, Millipore) with anti-FLAG (F3165, Sigma) and anti-HA (sc-7392x, Santa Cruz), and the co-immunoprecipitated DNA was recovered and analysed by quantitative PCR (qPCR) with SYBR Premix ExTaq Mix (DRR041A, TaKaRa Bio). For ChIP–reChIP assays, *nf-yc9 rgl2 pNF-YC9:NF-YC9-3FLAG pRGL2:RGL2-6HA* and *pRGL2:RGL2-6HA* seeds were incubated under 5 μM PAC for 12 h and harvested for fixation. The sonicated chromatins were immunoprecipitated by anti-HA agarose conjugate (the first ChIP), and then washed by the ChIP buffer and eluted with 10 mM dithiothreitol (DTT). The eluted chromatins were diluted 20-fold with dilution buffer (1% Triton X-100, 2 mM EDTA, 20 mM Tris-HCl at pH 8.1, 150 mM NaCl) and immunoprecipitated again by anti-FLAG (the second ChIP). The recovered DNA was purified and quantified by qPCR. Relative enrichment fold was calculated by normalizing the amount of a target DNA fragment against that of a genomic fragment of a reference gene *TUB8*, and then against the respective input DNA samples. The enrichment of a *PP2A* genomic fragment was used as the negative control. The primers used are listed in Supplementary Table 1.

### Transient expression assay

To generate the *ABI5:GUS* reporter construct, ∼1.8 kb *ABI5* promoter was cloned into HY107 containing *GUS* gene^36^. The *mABI5:GUS* constructs carrying mutated CCAAT elements were generated by overlapping PCR reaction from the *ABI5:GUS* construct. The *35S:NF-YC9-6HA* and various *DELLA* constructs were used as effectors, and a construct containing the firefly luciferase driven by 35S promoter in pGreen-35S was used as an internal control to evaluate the protoplast transfection efficiency. All the primers used for transient expression assay are listed in Supplementary Table 1. *Arabidopsis* mesophyll protoplasts were prepared, transfected and cultured for 24 h before protein extraction and detection. Activities of GUS and luciferase were measured using microfluorometer (Cary Eclipse, USA). Relative GUS activity was calculated by normalizing against the luciferase activity, and the data presented were the averages of three biological replicates.

### Protein–DNA affinity pull-down assay

The coding regions of yeast *NF-YA* (158-214aa) and yeast *NF-YB* (35-127aa) core domains for NF-Y combination^67^ were cloned into the pQE30 vector to produce fusion proteins of His-Yeast NF-YA core and His-Yeast NF-YB, respectively. Primers used for constructions are listed in Supplementary Table 1. His-NF-YC9 and GST-RGL2 fusion proteins were induced and purified as described above. Protein–DNA affinity assay was conducted with a modified method^68^. 1 μg of His-NF-YC9, 1 μg of His-Yeast NF-YA and 1 μg of His-Yeast NF-YB proteins were pre-incubated with various DNA fragments (0.5 μg each) produced by PCR using specific primes (Supplementary Table 1) in the binding buffer (100 mM phosphate, 150 mM NaCl and 1 mM EDTA, pH 7.5) at 4 °C for 4 h. Then, the incubated protein–DNA samples were mixed with GST or GST-RGL2 immobilized by Glutathione Sepharose Beads (17-0756-01, Amersham Biosciences) at 4 °C for additional 4 h. After being washed with binding buffer three times, proteins and DNA retained on the beads were subsequently eluted by elution buffer (2% SDS, 50 mM Tris-HCl, 150 mM NaCl and 1 mM EDTA, pH 8.0) at 65 °C for 15 min twice, and the DNA pulled down was extracted and quantified by qPCR using specific primers (Supplementary Table 1). The protein–DNA binding efficiency was calculated by normalizing the amount of DNA precipitated by proteins against that of the respective input DNA, and *PP2A* genomic DNA fragment was amplified and used as an internal control in this assay. The precipitated proteins bound to beads were detected by anti-His or anti-GST antibody as described above. Uncropped scans of western blot results are shown in Supplementary Fig. 16.

### Data availability

The RNA-seq data used in this study have been deposited in the Gene Expression Omnibus database under accession number GSE84753. All other data supporting the findings of this study are available within the article and its Supplementary Information files or on request from the corresponding author.

## Additional information

**How to cite this article:** Liu, X. *et al*. The NF-YC–RGL2 module integrates GA and ABA signalling to regulate seed germination in *Arabidopsis*. *Nat. Commun.* 7:12768 doi: 10.1038/ncomms12768 (2016).

## Supplementary Material

Supplementary Information Supplementary Figures 1-16 and Supplementary Table 1.

Supplementary Data 1 Summary of RNA-seq datasets

Supplementary Dataset 2 List of PAC, NF-YC and RGL2 regulated genes

Supplementary Data 3 List of co-regulated genes by PAC, NF-YCs, and RGL2
