# Supplementary material for: The NF-YC–RGL2 module integrates GA and ABA signalling to regulate seed germination in Arabidopsis
Source: Nat Commun. 2016 Sep 14;7:12768. doi: 10.1038/ncomms12768 (PMC5027291; doi:10.1038/ncomms12768)
Supplement: Supplementary Information — Supplementary Figures 1-16 and Supplementary Table 1. [file ncomms12768-s1.pdf]

SUPPLEMENTARY INFORMATION

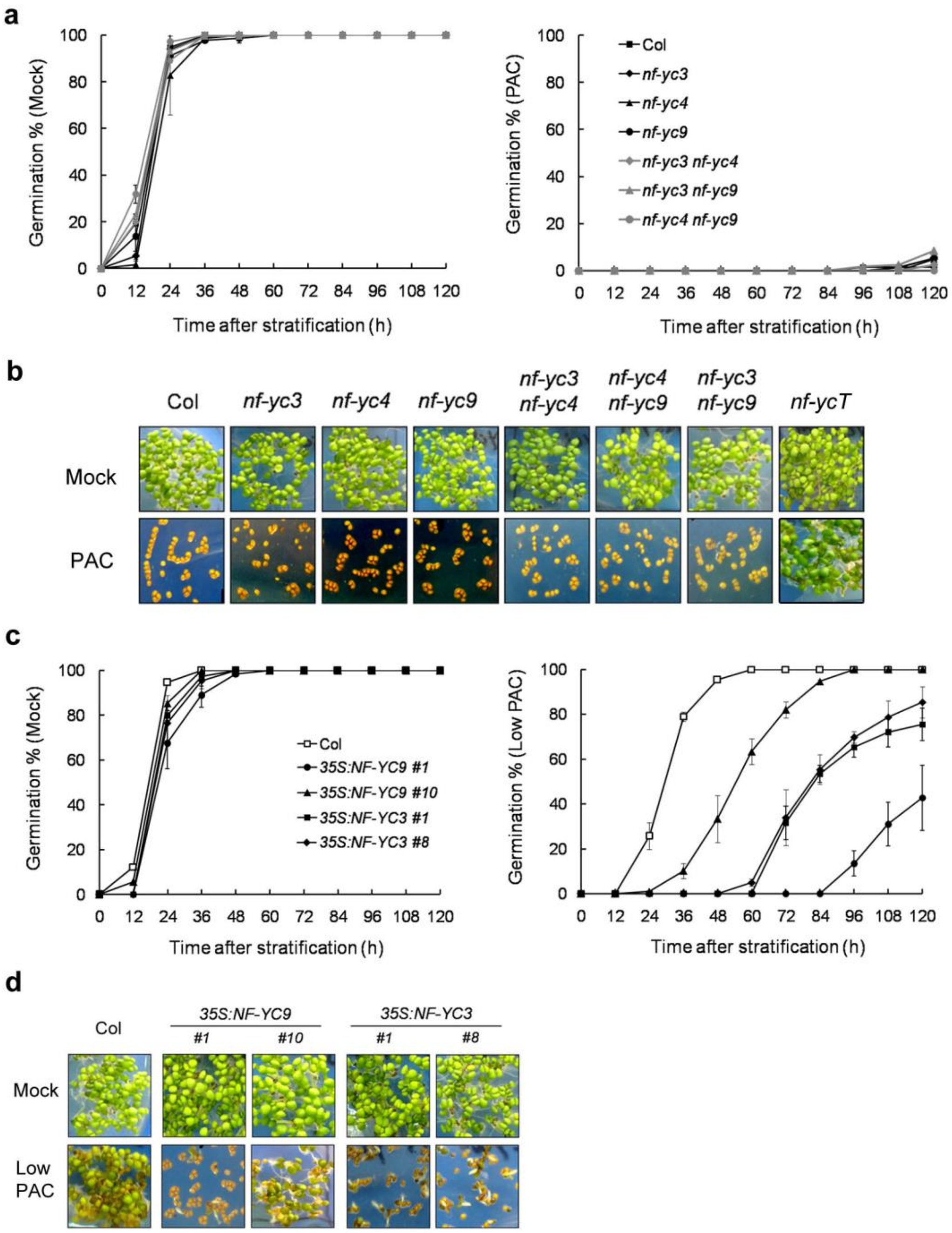

Supplementary Figure 1. The PAC sensitivity of *NF-YC* mutants and *NF-YC* overexpression lines compared with the wild type during seed germination. (**a**) Seeds of *nf-yc3*, *nf-yc4*, *nf-yc9*, *nf-yc3 nf-yc4*, *nf-yc3 nf-yc9*, *nf-yc4 nf-yc9*, and the wild-type (Col) were grown on 1/2 MS medium containing either 5 µM PAC or mock. The germination rate of all genotypes was recorded at every 12 h until 120 HAS (hour after stratification). (**b**) Germination phenotypes of the seeds described in (**a**) were observed at 120 HAS. (**c**) Seeds of *35S:NF-YC3#1*, *35S:NF-YC3#8*, *35S:NF-YC9#1*,

35S:NF-YC9#10 independent overexpressing lines, and the wild-type were grown on 1/2 MS medium containing either 0.5 μM PAC (low PAC) or mock. The germination rates of all genotypes were recorded at every 12 h until 120 HAS. (**d**) Germination phenotypes of the seeds described in (**c**) were observed at 120 HAS. All above data represent mean ±SD of at least 100 seeds.

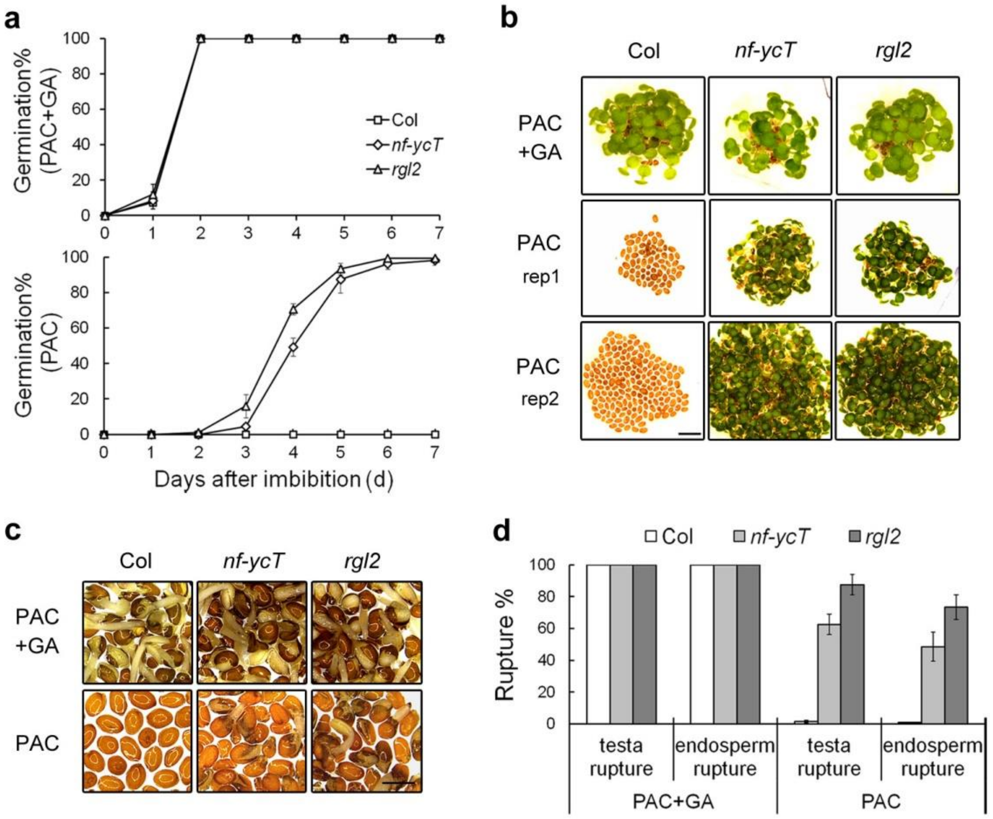

Supplementary Figure 2. Germination phenotypes of *nf-ycT* and *rgl2* mutants in absence of stratification. (**a**) Statistic analysis of germination rate in *nf-ycT*, *rgl2*, and the wild-type (Col) seeds on 1/2 MS medium containing PAC (5 µM) or PAC+GA (10 µM), respectively, in absence of stratification. The germination rate of all genotypes was recorded every 1 d until 7 d after imbibition. (**b**) Germination phenotypes of *nf-ycT*, *rgl2*, and Col seeds were observed at 7 d after imbibition under two replicates of PAC treatment (PAC rep1 and PAC rep2) and PAC+GA, respectively. Scale bar = 2 mm. (**c**) Testa and endosperm ruptures of *nf-ycT*, *rgl2*, and Col seeds were observed at 4 d after imbibition under PAC (5 µM) or PAC+GA (10 µM), respectively. Scale bar = 1 mm. (**d**) Statistic analysis of the testa and endosperm ruptures of the seeds described in (**c**). Data represent mean ±SD of at least 100 seeds.

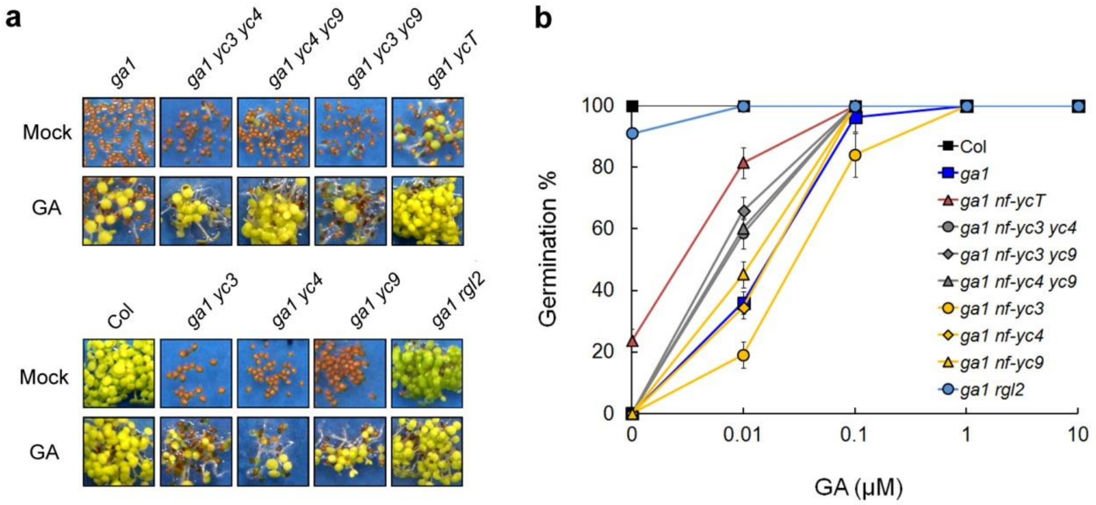

Supplementary Figure 3. Germination observations of various *NF-YC* mutants in *ga1* background under GA treatment. (**a**) Germination phenotypes of *ga1 nf-yc3*, *ga1 nf-yc4*, *ga1 nf-yc9*, *ga1 nf-yc3 nf-yc4*, *ga1 nf-yc3 nf-yc9*, *ga1 nf-yc4 nf-yc9*, *ga1 nf-ycT*, *ga1* and the wild-type (Col) seeds were observed at 120 HAS on 1/2 MS medium containing either 0.01 µM GA or mock. (**b**) Statistic analysis of germination rate in seeds described in (**a**) in response to different concentrations of GA, and germination rates were recorded at 120 HAS. Data represent mean ±SD of at least 50 seeds.

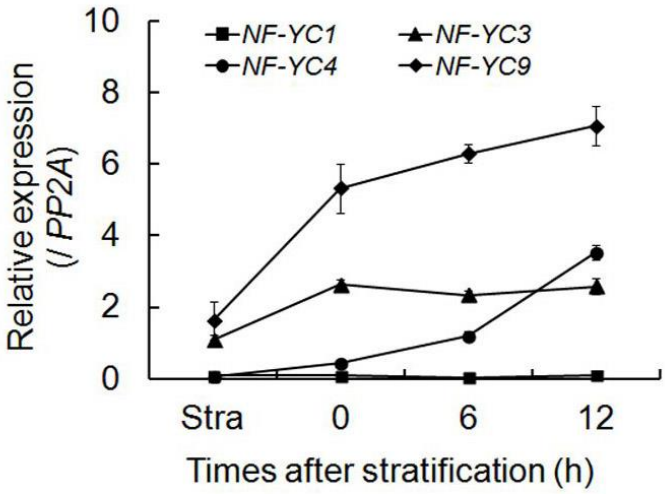

Supplementary Figure 4. The time-course expression pattern of four *Arabidopsis NF-YC* homologous genes during seed germination. The wild-type seeds were grown on 1/2 MS medium and total RNA was extracted according to the indicated time for quantitative RT-PCR analysis of *NF-YC1*, *NF-YC3*, *NF-YC4*, and *NF-YC9* expression. *PP2A* was amplified as an internal control. Data represent mean ± SD of three replicates.

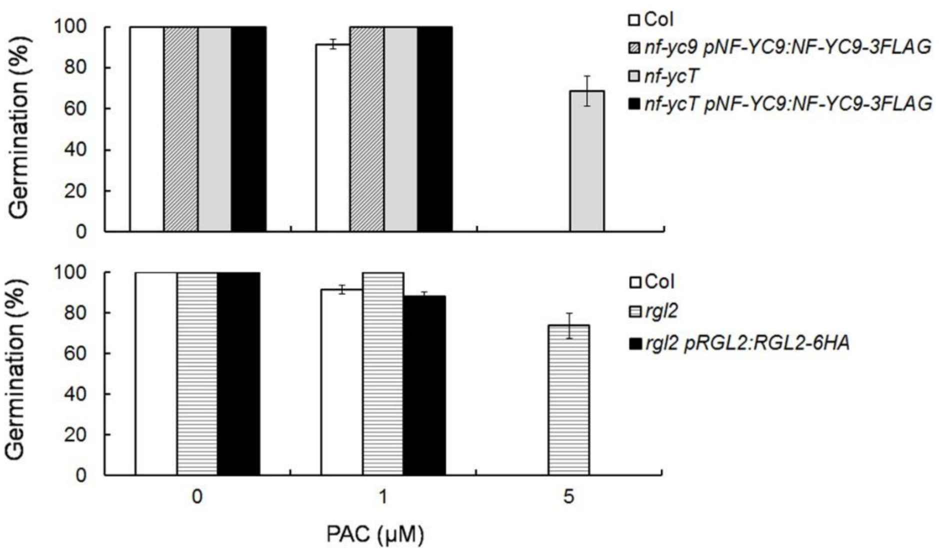

Supplementary Figure 5. *pNF-YC9:NF-YC9-3FLAG* fully rescues the PAC insensitivity of *nf-ycT* seeds. The germination rates of *nf-ycT*, *nf-yc9 pNF-YC9:NF-YC9-3FLAG*, *nf-ycT pNF-YC9:NF-YC9-3FLAG*, *rgl2*, *rgl2 pRGL2:RGL2-6HA*, and the wild-type seeds in response to different concentrations of PAC were recorded at 96 HAS. Data represent mean ± SD of three replicates.

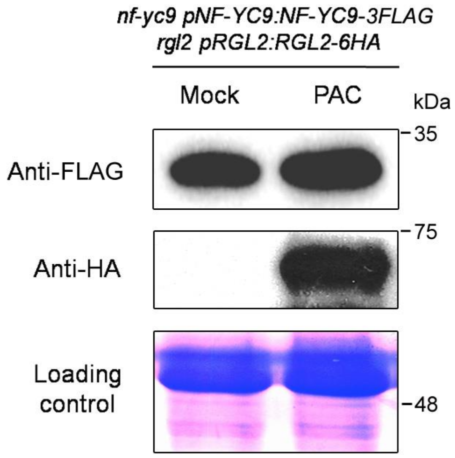

Supplementary Figure 6. The immune detection of NF-YC9-3FLAG and RGL2-6HA in *nf-yc9 pNF-YC9:NF-YC9-3FLAG rgl2 pRGL2:RGL2-6HA*. Seeds were grown on 1/2 MS medium containing 5 µM PAC or not and collected for protein extraction at 12 HAS.

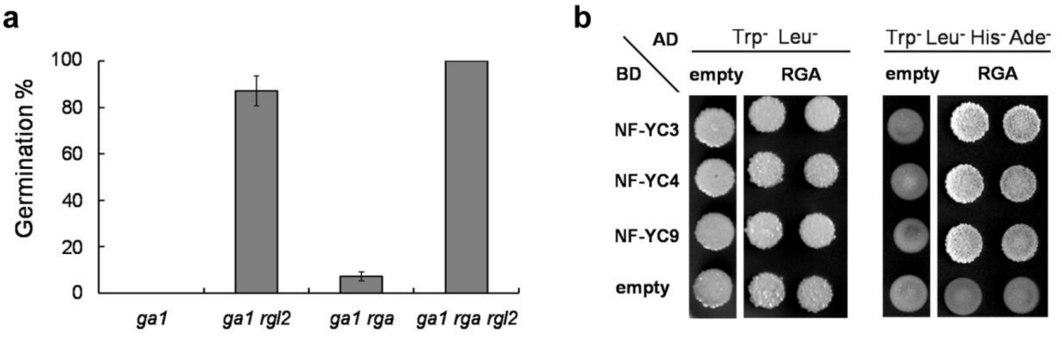

Supplementary Figure 7. Both of DELLA proteins *RGA* and *RGL2* play important roles in seed germination. (**a**) *rga rgl2* fully rescues the non-germination phenotype of *ga1*. Seeds of *ga1 rga-28*, *ga1 rgl2*, *ga1 rga-28 rgl2*, and *ga1* were grown on 1/2 MS medium, and germination rates were recorded at 120 HAS. Data represent mean ±SD of at least 100 seeds. (**b**) Yeast two-hybrid assays show the interactions between NF-YCs and RGA. Transformed yeast cells were grown on SD/-Trp/-Leu and SD/-Trp/-Leu/-His/-Ade medium.

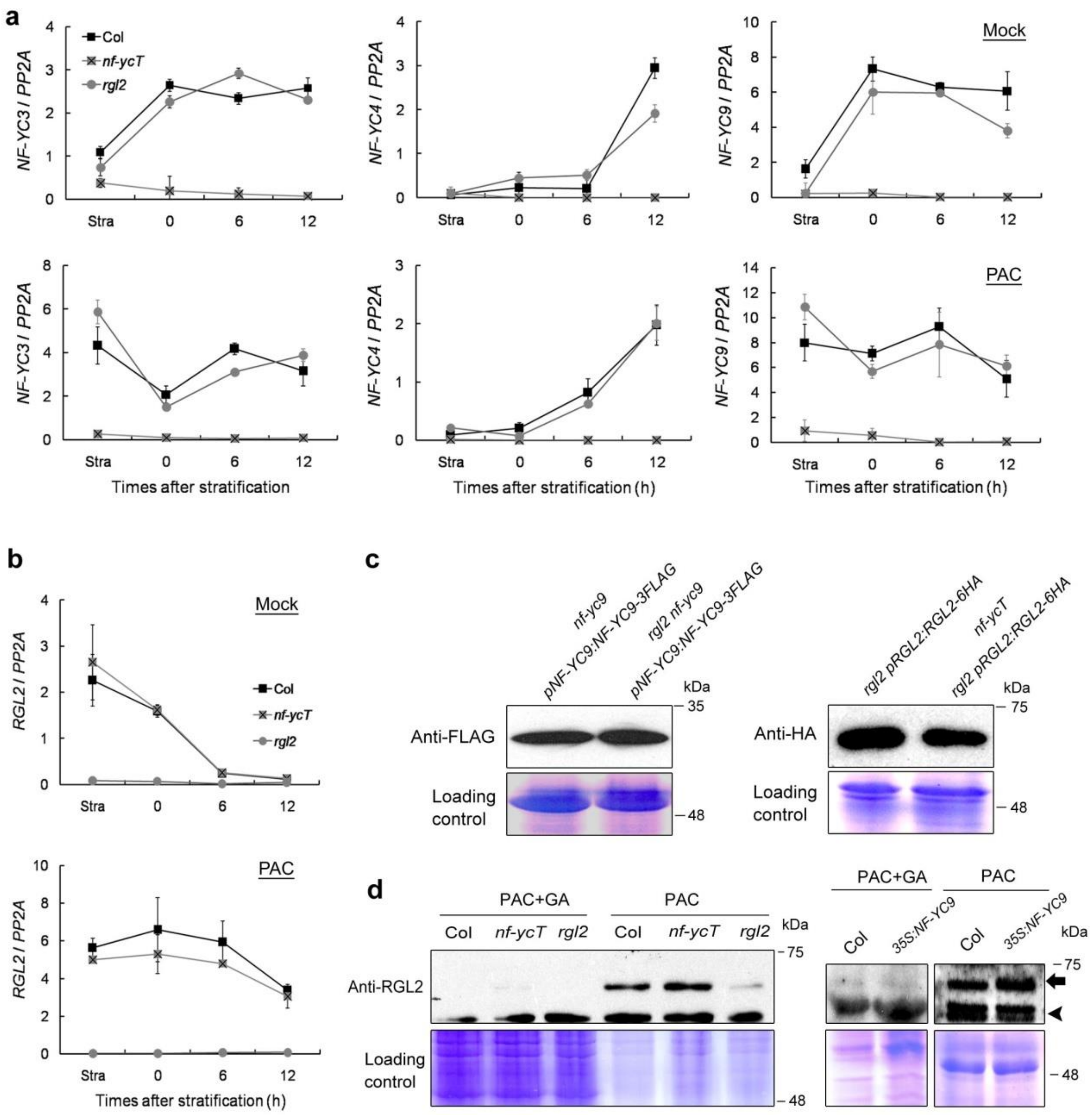

Supplementary Figure 8. The expression pattern analysis of *NF-YCs* and *RGL2* in *nf-ycT*, *rgl2* and the wild-type germinating seeds. (**a**) Quantitative RT-PCR analysis of *NF-YC3*, *NF-YC4*, and *NF-YC9* expression. Seeds were grown on 1/2 MS medium containing 5 μM PAC or not and total RNA was extracted at 1.5 d stratification (Stra), and 0, 6, 12 HAS, respectively. *PP2A* was amplified as an internal control. Data represent mean ± SD of three replicates. (**b**) Quantitative RT-PCR analysis of *RGL2* expression. Seeds were grown on 1/2 MS medium containing 5 μM PAC or not and total RNA was extracted at 1.5 d stratification (Stra), and 0, 6, 12 HAS, respectively. *PP2A* was amplified as an internal control. Data represent mean ± SD of three replicates. (**c**) Loss of *NF-YCs* or *RGL2* rarely affects the abundance of RGL2 or NF-YC9 proteins. The immune detection of NF-YC9-3FLAG and RGL2-6HA was performed using *nf-yc9 pNF-YC9:NF-YC9-3FLAG*, *rgl2 nf-yc9 pNF-YC9:NF-YC9-3FLAG*, *rgl2 pRGL2:RGL2-6HA*, and *nf-ycT rgl2 pRGL2:RGL2-6HA* lines. (**d**) The immune detection of endogenous RGL2 protein

levels in the wild type, *nf-ycT*, *rgl2*, and *35S:NF-YC9* under PAC or PAC+GA treatment by RGL2 antibody (AS11 1803, Agrisera). For RGL2 analysis in *nf-ycT* and *rgl2*, seeds were grown on 1/2 MS medium containing 5 µM PAC and PAC+GA (10 µM) and collected at 12 HAS. For RGL2 analysis in *35S:NF-YC9*, seeds were grown on 1/2 MS medium containing 0.5 µM PAC and PAC+GA (10 µM) and collected at 12 HAS. Arrow indicates the specific bands of RGL2, while arrowhead indicates the non-specific bands.

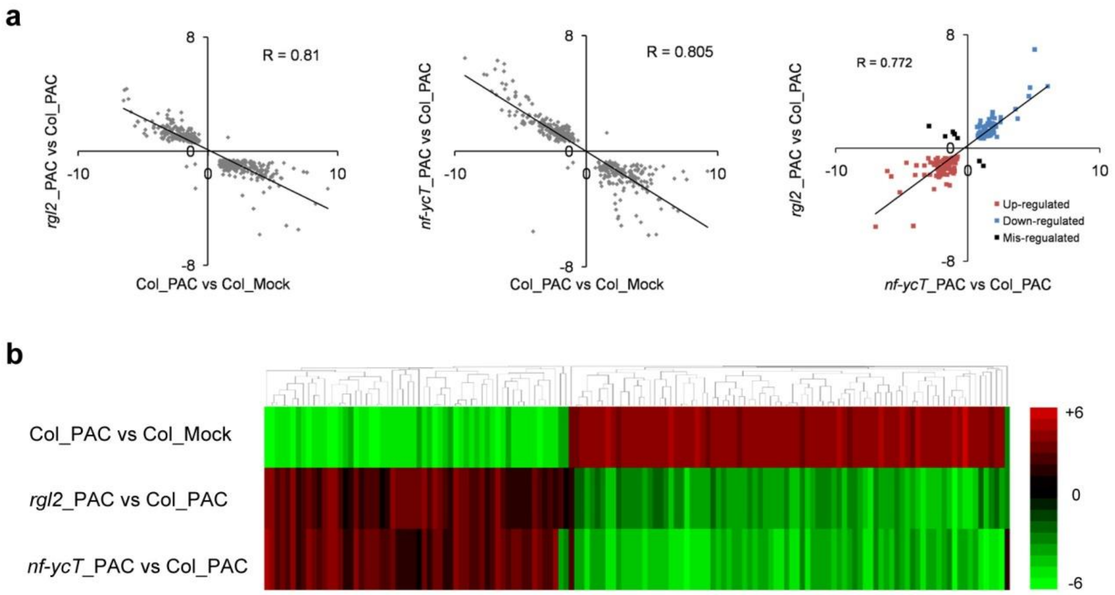

Supplementary Figure 9. Correlation and clustering analysis of expression regulatory pattern between coregulated genes by NF-YCs, RGL2 and GA. (**a**) Left panel indicates the scatter plot of log2-fold change values for 444 RGL2 and GA coregulated genes in "*rgl2*_PAC vs Col_PAC" versus "Col_PAC vs Col_Mock"; Middle panel indicates the scatter plot of log2-fold change values for 374 NF-YCs and GA coregulated genes in "*nf-ycT*_PAC vs Col_PAC" versus "Col_PAC vs Col_Mock"; Right panel indicates the scatter plot of log2-fold change values for 174 NF-YCs and RGL2 coregulated genes in "*nf-ycT*_PAC vs Col_PAC" versus "*rgl2*_PAC vs Col_PAC". (**b**) Heat map of 142 co-regulated genes by GA, NF-YCs, and RGL2. The scale bar indicates fold changes (log2 value).

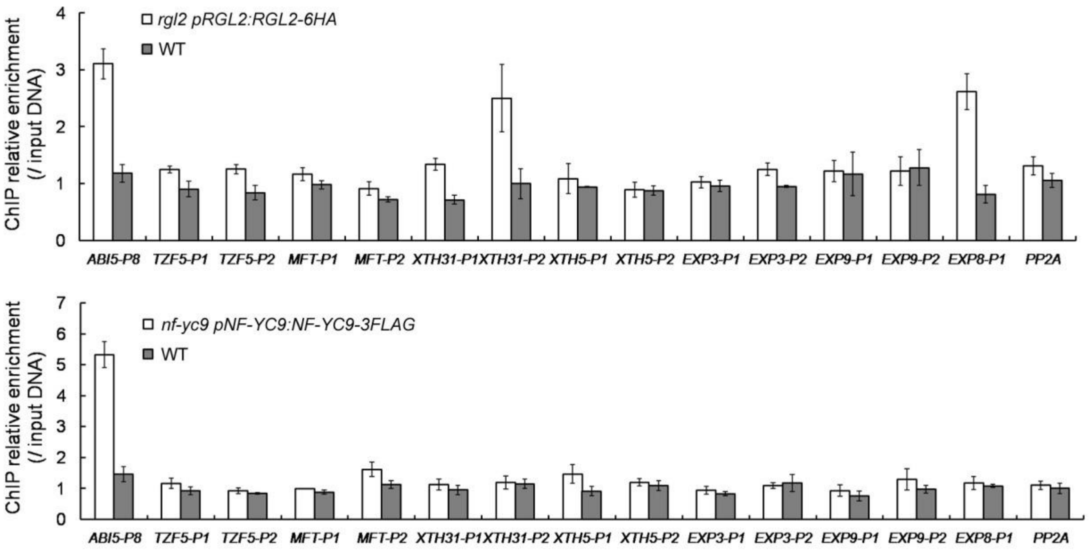

Supplementary Figure 10. ChIP analysis of NF-YC9 and RGL2 binding to the representative co-regulated genes. *nf-yc9 pNF-YC9:NF-YC9-3FLAG*, *rgl2 pRGL2:RGL2-6HA*, and the wild-type seeds were grown on 1/2 MS medium containing 5 µM PAC for 12 HAS harvested for ChIP assay. Relative enrichment fold was calculated by normalizing the amount of a target DNA fragment against that of a genomic fragment of a reference gene *TUB8*, and then against the respective input DNA samples. The enrichment of a *PP2A* genomic fragment was used as the negative control (the same below). *EXP8* served as a positive control of RGL2 binding (Stamm et al., 2012). Data represent mean ±SD of biological triplicates.

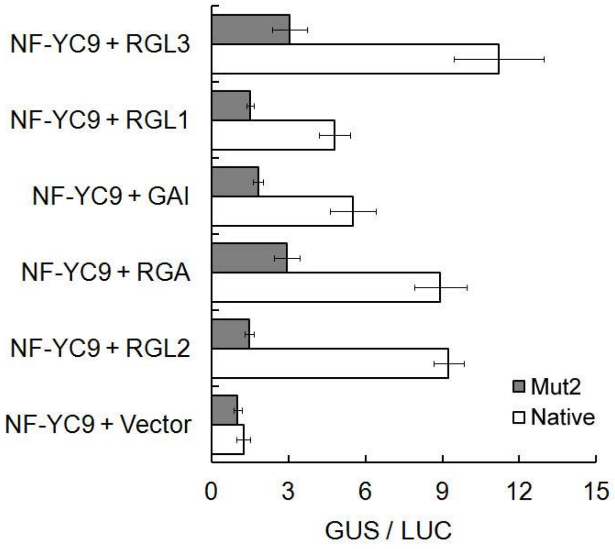

Supplementary Figure 11. Transient expression assay of *ABI5* promoter activity modulated by NF-YC9 and different DELLAs in *Arabidopsis* mesophyll protoplasts. Constructs used in transient expression assay are shown in Fig 5b. Data represent mean ± SD of three biological replicates.

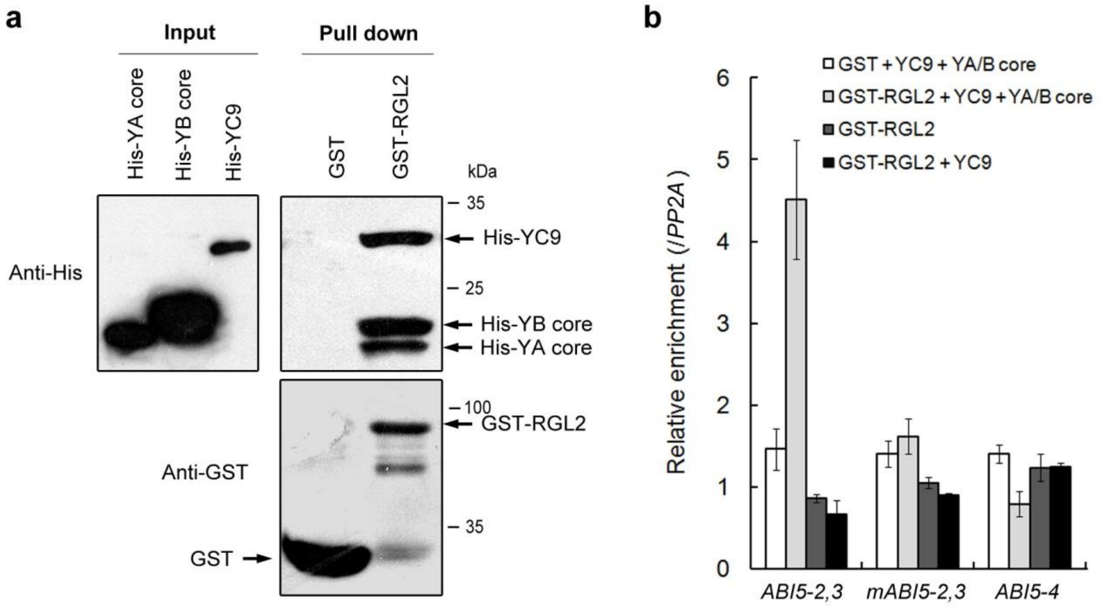

Supplementary Figure 12. Protein-DNA affinity pull-down assay showing that RGL2 recognizes *ABI5* promoter via binding to NF-Y complex. (**a**) Pull-down assay shows the direct interaction between GST-RGL2 and NF-Y complex of His-NF-YC9 (His-YC9), His-Yeast NF-YA core (His-YA core), and His-Yeast NF-YB core (His-YB core) proteins in vitro. His-NF-YA/B/C proteins were mixed with various DNA fragments indicated in (**b**) in advance and then incubated with immobilized GST or GST-RGL2 proteins. The immunoprecipitated fractions were detected by anti-His and anti-GST antibodies, respectively. Arrows indicate the specific bands of proteins detected. (**b**) qPCR analysis of pulled down DNA shows RGL2 binding affinity to DNA with different NF-YA/B/C protein combinations. *ABI5-2,3* indicates the *ABI5* promoter fragment DNA (-1363~-1057 bp) containing CCAAT-2 and -3. *mABI5-P23* indicates mutated *ABI5-P2,3* DNA in which CCAAT-2 and -3 were mutated. *ABI5-4* indicates the *ABI5* promoter fragment (-1752~-1575 bp) containing CCAAT-4 (Fig 5b). The relative DNA enrichment was calculated by normalizing the amount of DNA precipitated by proteins against that of the respective input DNA. *PP2A* genomic DNA fragment was amplified and used as an internal control. Data represent mean ± SD of three replicates.

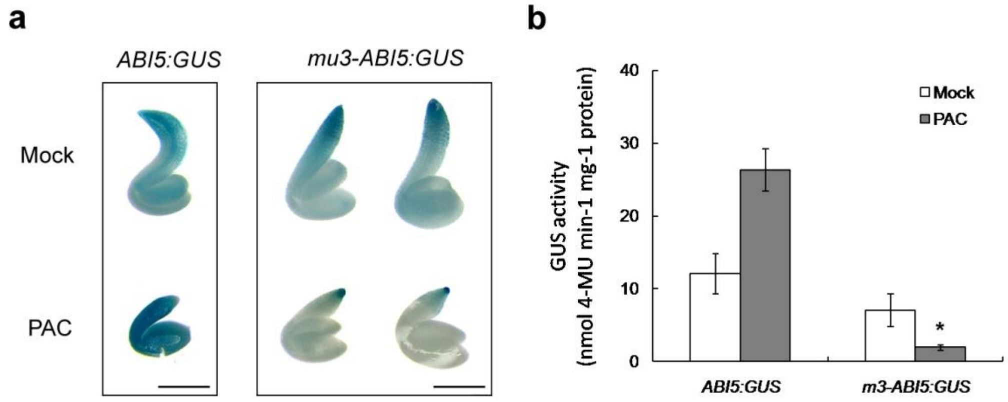

Supplementary Figure 13. Mutation of CCAAT-3 impairs the GUS expression in *ABI5:GUS* seeds. (**a**) Representative GUS staining of seeds harboring *ABI5:GUS* or its mutated version (mutated CCAAT-3) was photographed in seeds grown on 1/2 MS containing either 5 μM PAC or mock for 48 HAS. Scale bar = 0.5 mm. (**b**) Quantitative analysis of GUS activity in the transgenic seeds shown in (**a**). Data represent mean ± SD from at least 100 seeds of each genotype. Asterisk indicates significant changes in GUS activity between *ABI5:GUS* and *mu3-ABI5:GUS* seeds under PAC treatment (Student's *t*-test, $P<0.05$).

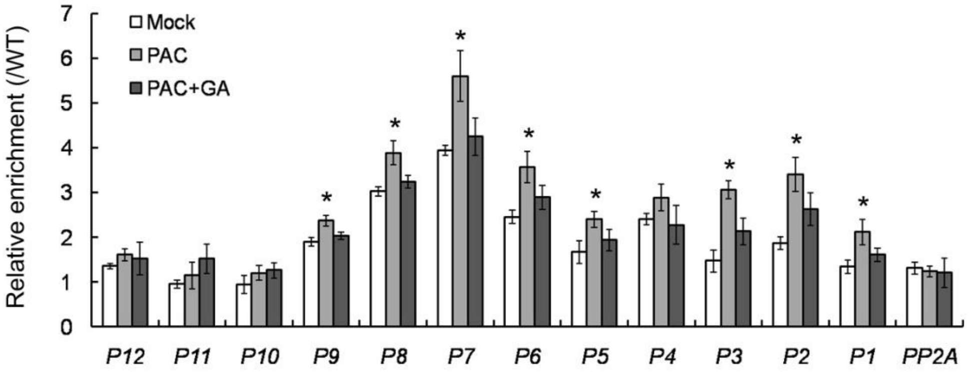

Supplementary Figure 14. GA abolishes the binding of NF-YC9 to the *ABI5* regulatory regions under PAC treatment. ChIP analysis of *nf-yc9 pNF-YC9:NF-YC9-3FLAG* was performed using the seeds grown on 1/2 medium containing 5 µM PAC, 5 µM PAC plus 1µM GA, or mock for 12 HAS. Data represent mean ± SD of three replicates. Asterisks indicate significant changes in enrichment fold between seeds with PAC and PAC+GA treatment (Student's *t*-test, $P<0.05$).

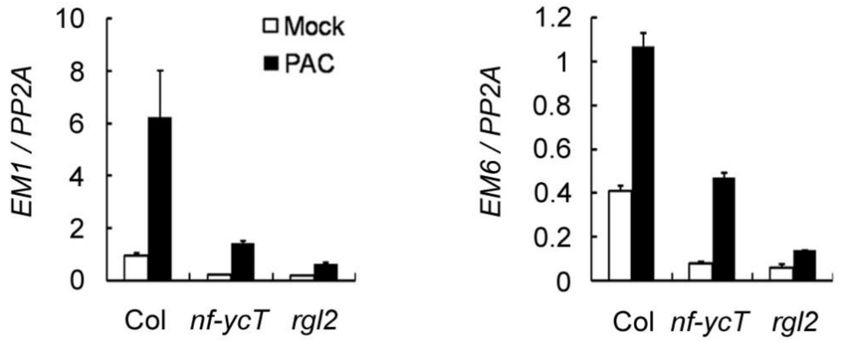

Supplementary Figure 15. Quantitative RT-PCR analysis of the expression level of *EM1* and *EM6* genes. The wild-type, *rgl2*, and *nf-ycT* seeds were grown on 1/2 MS medium containing either 5 μM PAC or mock for 12 HAS. The relative gene expression was normalized to that of *PP2A* internal. Data represent mean ± SD of three biological replicates.

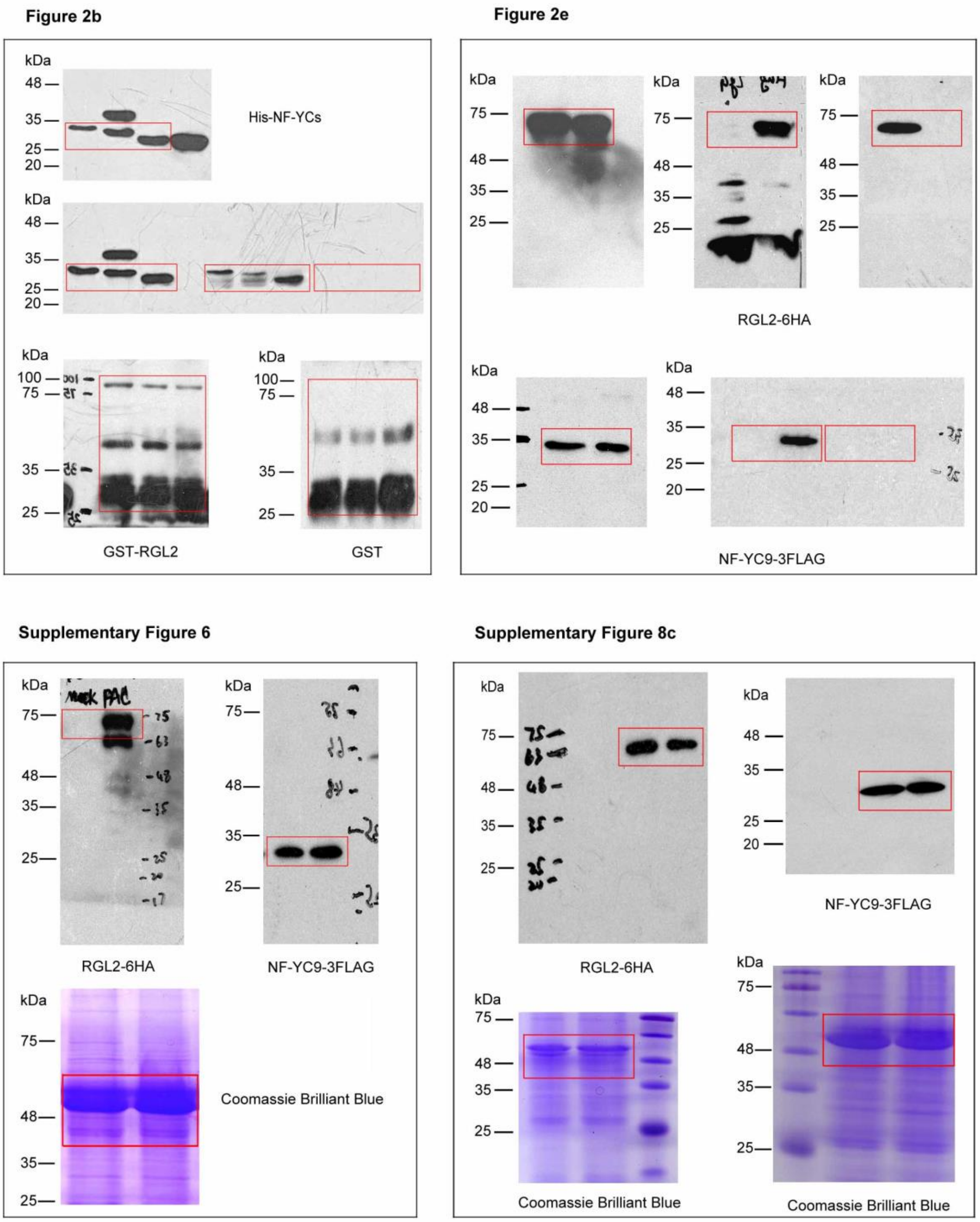

Supplementary Figure 16. Uncropped immunoblots used in main and supplementary figures. Red boxes highlight the areas shown in these figures.

Supplementary Figure 8d

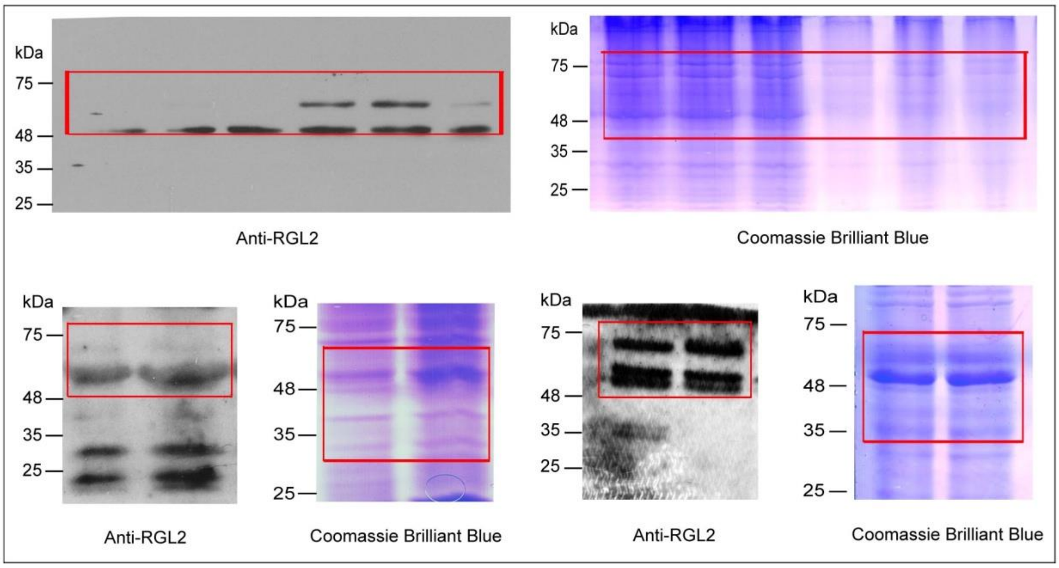

Supplementary Figure 12a

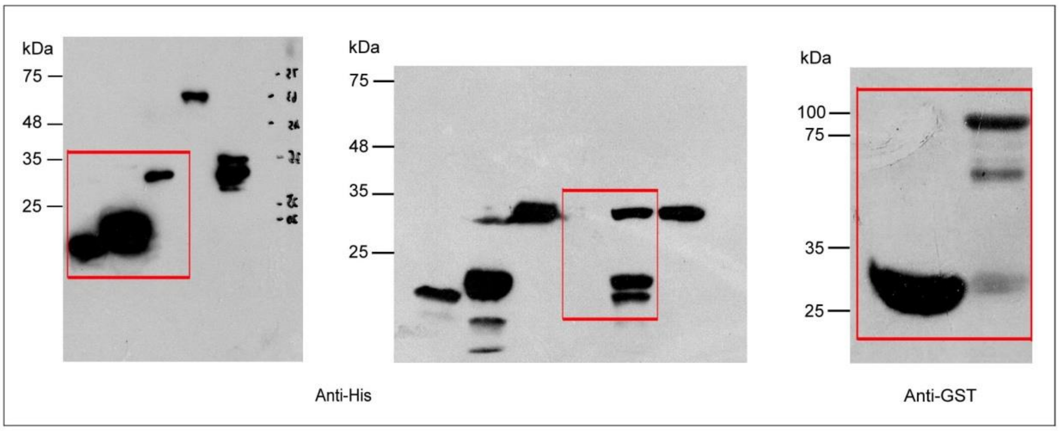

Supplementary Figure 16. Continued.

Supplementary Table 1. List of primers used in this study

Primers for quantitative RT-PCR

Gene name	Primers
ABI5	5'-CCAGTTGAAAGAAGAGAATGCGCAGC-3'<br>5'-AGTGGACAACTCGGGTTCCTCATCA-3'
TZF5	5'-TACAACAGAGTAGTCCTCTACGAT-3'<br>5'-CAGCCACCGAGAAATCGCTTA-3'
MFT	5'-CGAGCCGAACATGAGAGAAT-3'<br>5'-AAGTATCTCTTTTCCTCTTGAGGG-3'
EXP3	5'-TTAGGGTCGGCTTGTGG-3'<br>5'-CCGTATCCTTGGCTGTA-3'
EXP9	5'-ACTGGCAATCCAACGCACTTCT-3'<br>5'-TCAGACGCGGAAGTTCTTGC-3'
XTH5	5'-CACGTCGATGGATGTGAAGCT-3'<br>5'-CTTTCTGATCCCACCAACGTTT-3'
XTH31	5'-TGTCACTCTTTGGCTCG-3'<br>5'-ACCTCATCGTGGTCTCC-3'
EM1	5'-TCAAATGGTATGCGGTTATG-3'<br>5'-TATCACAAGTAAGACACGAAG-3'
EM6	5'-TGTCTCGTTTGTTTTCCAG-3'<br>5'-CACTATGTTGAGAATCCAC-3'
NF-YC1	5'-CAAAACCGACCAATGGATAC-3'<br>5'-GAGCTGTTGTTGTTGTTGTTG-3'
NF-YC3	5'-GATCAACAAGGACAATCATCA-3'<br>5'-TGGTAAGCTGGATGGTCTG-3'
NF-YC4	5'-AAGAGGAAGATGCAGCATCGG-3'<br>5'-CACATCATCACCACCACCAGC-3'
NF-YC9	5'-AAGTATTCCGAGGGGCACTG-3'<br>5'-CTAATTTTCCTGGTCAGGTTGG-3'
TIP41-like	5'-GTGAAAACTGTTGGAGAGAAGCAA-3'<br>5'-TCAACTGGATACCCTTTCGCA-3'
PP2A	5'-TATCGGATGACGATTCTTCGTGCAG-3'<br>5'-GCTTGGTCGACTATCGGAATGAGAG-3'

Primers for constructs in plant transformation

Construct name	Primers
35S:RGL2-6HA	5'-AGA ctgcag ATGAAGAGAGGATACGGAGAAAC-3'<br>5'-CGGcccgggGCGAGTTTCCACGCCGAGGT-3'
pRGL2:RGL2-6HA	5'-CCatcgatTCTCAACAGTCTCATGCCGAGATGATGG-3'<br>5'-AActgcagGGCGAGTTTCCACGCCGAGGT-3'

35S:RGA-6HA	5'-CAActgcagATGAAGAGAGATCATCACCAAT-3'<br>5'-CGGcccgggGTACGCCGCCGTCGAGAGTTTCCA-3'
35S:GAI-6HA	5'-CAAgtatacATGAAGAGAGATCATCATCATCA-3'<br>5'-CGGcccgggATTGGTGGAGAGTTTCCAAGCCGA-3'
35S:RGL1-6HA	5'-CAAgtatacATGAAGAGAGAGCACAACCACCG-3'<br>5'-CGGcccgggTTCCACACGATTGATTCGCCAC-3'
35S:RGL3-6HA	5'-AGActgcagATGAAACGAAGCCATCAAGAAACGT-3'<br>5'-CGGcccgggCCGCCGCAACTCCGCCGCTAGTTTCCA-3'
ABI5:GUS	5'-CAActgcagAGTTGCTGTAATCTTTAGGTCGCTGGT-3'<br>5'-GAAggatccCATTTAACAACTGCATCATATACAC-3'
mABI5:GUS	5'-TGAAGGTCAACATCTTTTGTCTAAAGTTATCTTTCGA**aCAta**GGA-3'<br>5'-GACCTGTCTAAGTTAGCATTCC**taTGt**TCGAAAGA-3'
mu3-ABI5:GUS	5'-TGGATTAGAAGAGCGAATTAAAGTGTGATCTTTTTTTACAT**taTGt**TTCATG-3'<br>5'-CGTCAATTTCACAAAACAAATCATGAA**aCAta**ATG-3'

Primers for constructs in yeast two-hybrid assay

Construct name	Primers
BD-NF-YC3	5'-GAGcatatgAGAGAAATGGATCAACAAGGA-3'<br>5'-AAAgaattcGCCAAGCTAATTGTCAGGA-3'
BD-NF-YC9	5'-AAAcatatgGATCAACAAGACCATGGACAG-3'<br>5'-GCGgaattcAGTTTCTTGCTAATTTTCCT-3'
BD-NF-YC4	5'-GGcatatgGACAATAACAACAACAACAAC-3'<br>5'-TAcccgggACCTTGGCTATCGAGATTACCA-3'
AD-RGL2	5'-CGCcatatgAAGAGAGGATACGGAGAAACA-3'<br>5'-TATcccgggTTTCAGGCGAGTCATCTCTAC-3'
AD-RGA	5'-CGAcccgggAATGAAGAGAGATCATCACCAATTC-3'<br>5'-ATTggtaccTCAGTGCGCCGCCGTCGAGAGTTTC-3'
AD-RGL2ΔG	5'-ATAcatatgATGAAGAGAGGATACGGAGAAAC-3'<br>5'-CGGcccgggGCACCACGATCCGAGTCGGAT-3'
AD-RGL2ΔD	5'-ATAcatatgGAATCGTCGGACGAGTCAACT-3'<br>5'-TATcccgggTTTCAGGCGAGTCATCTCTAC-3'
BD-NF-YC9 ΔC	5'-AAAcatatgGATCAACAAGACCATGGACAG-3'<br>5'-GCGgaattcCCGGGGAACAATATCCACAAGGA-3'
BD-NF-YC9 ΔN	5'-AAAcatatgCATCAGCAGCAGCAGCAGCAA-3'<br>5'-GCGgaattcAGTTTCTTGCTAATTTTCCT-3'
BD-NF-YC9 N	5'-AAAcatatgGATCAACAAGACCATGGACAG-3'<br>5'-GCGgaattcGATCTGGTGGAACGCCAGCT-3'
BD-NF-YC9 C	5'-AAAcatatgGAGGATCTCCGAGATGAAGTCT-3'<br>5'-GCGgaattcAGTTTCTTGCTAATTTTCCT-3'

BD-NF-YC9 HFD	5'-AAAcatatgCATCAGCAGCAGCAGCAGCAA-3'<br>5'-GCGgaattcCCGGGGAACAATATCCACAAGGA-3'

Primers for constructs in pull-down assay

Construct name	Primers
His-NF-YC3	5'-AATgagctcATGGATCAACAAGGACAATCAT-3'<br>5'-GCGgtcgacCTAATTGTCAGGATCCTGCTGCT-3'
His-NF-YC4	5'-AAgagctcATGGACAATAACAACAACAACAAC-3'<br>5'-TAcccgggACCTTGGCTATCGAGATTACCA-3'
His-NF-YC9	5'-AATggatccGATCAACAAGACCATGGAC-3'<br>5'-GCGgtcgacAGTTTCTTGCTAATTTTCCT-3'
GST-RGL2	5'-CGcccgggTATGAAGAGAGGATACGGAGAAAC-3'<br>5'-AAgcggccgcGGCGAGTTTCCACGCCGAGGT-3'

Primers for ChIP assay

Gene name	Primers
ABI5-P1	5'-GCTGAACAGGGACAAGTAACTG-3'<br>5'-TGGATACCACCTAAACGAC-3'
ABI5-P2	5'-CTTTGAGAGCATCAATTATCA-3'<br>5'-GTCCCTTATTCAACTATCA-3'
ABI5-P3	5'-CGAGTGGGTTAAGATATT-3'<br>5'-GGTAATTAAGTCGGTCCACGTGA-3'
ABI5-P4	5'-GGTAATTAAGTCGGTCCACGTGA-3'<br>5'-ATCATTAGCTTGGGTCATCA-3'
ABI5-P5	5'-GATCTTGGATTAGAAGACT-3'<br>5'-CAGATAAGTAAGGACCAGA-3'
ABI5-P6	5'-TTACCGCCTCCTACCCAT-3'<br>5'-CTGAGAGAATCCGCTTCT-3'
ABI5-P7	5'-CTTTGTCTCTGATCATGGGCCT-3'<br>5'-GAAGAGAGGCGTGAAGGTCA-3'
ABI5-P8	5'-AGATCACACTTTAATTCGCTCT-3'<br>5'-ACGTGTGACTTCGGCTACA-3'
ABI5-P9	5'-GTCTCACTAAACGTAATTCTA-3'<br>5'-TCGTTGAGCTTCTAGCTGGTG-3'
ABI5-P10	5'-TAGGTCGCTGGTTCGATTC-3'<br>5'-AGTGCACGTGGACTATTCACT-3'
ABI5-P11	5'-CAGCTGCAGGTTCACATTCTG-3'<br>5'-CACCCTCGCCTCCATTGTTAT-3'
ABI5-P12	5'-CCAGTTGAAAGAAGAGAATGCGCAGC-3'<br>5'-TCTCCAACTCCGCCTTGATGA-3'

TZF5-P1	5'-ATGGATGTATCCACTAACGCA-3'<br>5'-ATGGATGTATCCACTAACGCA-3'
TZF5-P2	5'-GCGTTATTTCTTCATAACTATGCGTGT-3'<br>5'-TATTGGTTACGTGGCTCGCATGG-3'
MFT-P1	5'-AGAGGAAATTATCGCCAACGT-3'<br>5'-ATGGGCATCTATGTCATGGTT-3'
MFT-P2	5'-ATGATCCTCCTAACCGACGGC-3'<br>5'-AACCTATAGGCCATGCATAGG-3'
XTH31-P1	5'-CCGTACGGTAAGTAGCT-3'<br>5'-CATTTGAAGTTGGTTCAACA-3'
XTH31-P2	5'-GACATGGGATCAATCCA-3'<br>5'-CCTTTTGAGTGGTTACTACA-3'
XTH5-P1	5'-TCCCTAAGTTACTGCTGCCA-3'<br>5'-CATTCTCACTCTGATCACAC-3'
XTH5-P2	5'-GCCATCCTCTTGAAACTTGA-3'<br>5'-TACCACCATATTTGCTTGG-3'
EXP3-P1	5'-ATTGCCACCTTCGGTTTAGT-3'<br>5'-AGAAAGCGGGAAGGACTACG-3'
EXP3-P2	5'-CTGCAAATGATTTGCTATACAT-3'<br>5'-ACTTCCAACTTCATATGTAGAAACT-3'
EXP9-P1	5'-TCCCTAAGTTACTGCTGCCA-3'<br>5'-CATTCTCACTCTGATCACAC-3'
EXP9-P2	5'-GCCATCCTCTTGAAACTTGA-3'<br>5'-TACCACCATATTTGCTTGG-3'
EXP8-P1	5'-AGAAAGCGGGAAGGACTACG-3'<br>5'-TTTGTTGAGGAGGGGTTAG-3'
TUB8	5'-CCGTTTCAAATTCTCTCTCTC-3'<br>5'-CAAACTACTTCCCAGAACTTAGC-3'
PP2A	5'-TATCGGATGACGATTCTTCGTGCAG-3'<br>5'-GCTTGGTCGACTATCGGAATGAGAG-3'

Primers for constructs in BiFC assay

Construct name	Primers
YFP^{C}*-NF-YC9*<br>*YFP*^{N}*-NF-YC9*<br>*NF-YC9-YFP*^{C}<br>*NF-YC9-YFP*^{N}	5'-TTTctcgagGAATGGATCAACAAGACCATGGAC-3'<br>5'-AATggatccAATTTTCCTGGTCAGGTTGGTCA-3'
YFP^{C}*-RGL2*<br>*YFP*^{N}*-RGL2*<br>*RGL2-YFP*^{C}<br>*RGL2-YFP*^{N}	5'-AGActgcagATGAAGAGAGGATACGGAGAAAC-3'<br>5'-CGGcccgggGCGAGTTTCCACGCCGAGGT-3'

Primers for constructs in GUS assay

Probe name	Oligo sequences
Mut1	5'-AGCAATAAATAAACTAATAATTTACTCTT**aCAta**AAAG-3'
	5'-GATATTTTCCTCTTTTGGTAACGAAAACTTT**taTGt**AAGAGT-3'
Mut2	5'-TGAAGGTCAACATCTTTTGTCTAAAGTTATCTTTCGA**aCAta**GGA-3'
	5'-GACCTGTCTAAGTTAGCATTCC**taTGt**TCGAAAGA-3'
Mut3	5'-TGGATTAGAAGAGCGAATTAAAGTGTGATCTTTTTTTACAT**taTGt**TTCATG-3'
	5'-CGTCAATTTCACAAAACAAATCATGAA**aCAta**ATG-3'
Mut4	5'-CC**taTGt**GAGTGCACGTGGACTATTCACT-3'
	5'-CACTC**aCAta**GGAAGTTCGGAATCATGTTTTTTTTTTTTTGTCA-3'

Primers for protein-DNA affinity pull-down assay

Name	Primers
ABI5-2,3	5'-GTTAGCATTCCATTGGTCGAAAGATAACTT-3'
(for DNA fragment PCR)	5'-TTTTTTTACATATTGGTTCATGATTTGTTT-3'
mABI5-2,3	5'-GTTAGCATTCCtaTGtTCGAAAGATAACTT-3'
(for DNA fragment PCR)	5'-TTTTTTTACATtaTGtTTCATGATTTGTTT-3'
ABI5-4	5'-GGCTATTAGAAACACTTGATA-3'
(for DNA fragment PCR)	5'-AAAGTCGAGAAATTATCCTCTTCCT-3'
ABI5-2,3	5'-TAATTCGCTCTTCTAATCCA-3'
(for qPCR)	5'-TGACTTCGGCTACACAAATGT-3'
mABI5-2,3	5'-TAATTCGCTCTTCTAATCCA-3'
(for qPCR)	5'-TGACTTCGGCTACACAAATGT-3
ABI5-4	5'-AGTCCTTATGCAGTGAATAGT-3'
(for qPCR)	5'-CAAAGTCGAGAAATTATCCTCT-3'
PP2A	5'-TATCGGATGACGATTCTTCGTGCAG-3'
(for DNA fragment PCR and qPCR)	5'-GCTTGGTCGACTATCGGAATGAGAG-3'
His-Yeast NF-YA core	5'-CAAggatccCAAAGCGATGTTTTAGGAACCG-3'
(for prokaryotic expression)	5'-TAAcccgggTCAGAACCTCCCACCTTCACCACGA-3'
His-Yeast NF-YB core	5'-CAAggatccCTAAGAGAGCAGGACAGATGGCT-3'
(for prokaryotic expression)	5'-TAAcccgggTCATTGTTGCCTGTATTTAGCCAAGT-3'
ABI5-2,3	5'-GTTAGCATTCCATTGGTCGAAAGATAACTT-3'
(for DNA fragment PCR)	5'-TTTTTTTACATATTGGTTCATGATTTGTTT-3'
mABI5-2,3	5'-GTTAGCATTCCtaTGtTCGAAAGATAACTT-3'
(for DNA fragment PCR)	5'-TTTTTTTACATtaTGtTTCATGATTTGTTT-3'
ABI5-4	5'-GGCTATTAGAAACACTTGATA-3'
(for DNA fragment PCR)	5'-AAAGTCGAGAAATTATCCTCTTCCT-3'
ABI5-2,3	5'-TAATTCGCTCTTCTAATCCA-3'
(for qPCR)	5'-TGACTTCGGCTACACAAATGT-3'
mABI5-2,3	5'-TAATTCGCTCTTCTAATCCA-3'
(for qPCR)	5'-TGACTTCGGCTACACAAATGT-3
